# Biocompatibility and degradation comparisons of four biodegradable copolymeric osteosynthesis systems used in maxillofacial surgery: A goat model with four years follow-up

**DOI:** 10.1016/j.bioactmat.2022.01.015

**Published:** 2022-01-19

**Authors:** Barzi Gareb, Nico B. van Bakelen, Léon Driessen, Pieter Buma, Jeroen Kuipers, Dirk W. Grijpma, Arjan Vissink, Ruud R.M. Bos, Baucke van Minnen

**Affiliations:** a University of Groningen, University Medical Center Groningen, Department of Oral and Maxillofacial Surgery, Hanzeplein 1, 9713 GZ, P.O. Box 30001, 9700 RB, Groningen, the Netherlands; b Radboud University Medical Center, Radboud Institute for Molecular Life Sciences, Orthopaedic Research Lab, Houtlaan 4, 6525 XZ, P.O. Box 9102, 6500 HC, Nijmegen, the Netherlands; c University of Groningen, University Medical Center Groningen, Department of Biomedical Sciences of Cells and Systems, Hanzeplein 1, 9713 GZ, P.O. Box 30001, 9700 RB, Groningen, the Netherlands; d University of Twente, TechMed Centre, Department of Biomaterials Science and Technology, Drienerlolaan 5, 7522 NB, P.O. Box 217, 7500 AE, Enschede, the Netherlands

**Keywords:** Biocompatible materials, Absorbable implants, Polymers, Orthopaedic fixation devices, Reconstructive surgical procedures, Fracture fixation

## Abstract

Applying biodegradable osteosyntheses avoids the disadvantages of titanium osteosyntheses. However, foreign-body reactions remain a major concern and evidence of complete resorption is lacking. This study compared the physico-chemical properties, histological response and radiographs of four copolymeric biodegradable osteosynthesis systems in a goat model with 48-months follow-up. The systems were implanted subperiosteally in both tibia and radius of 12 Dutch White goats. The BioSorb FX [poly(70LLA-*co*-30DLLA)], Inion CPS [poly([70–78.5]LLA-*co*-[16–24]DLLA-*co*-4TMC)], SonicWeld Rx [poly(DLLA)], LactoSorb [poly(82LLA-*co*-18GA)] systems and a negative control were randomly implanted in each extremity. Samples were assessed at 6-, 12-, 18-, 24-, 36-, and 48-month follow-up. Surface topography was performed using scanning electron microscopy (SEM). Differential scanning calorimetry and gel permeation chromatography were performed on initial and explanted samples. Histological sections were systematically assessed by two blinded researchers using (polarized) light microscopy, SEM and energy-dispersive X-ray analysis. The SonicWeld Rx system was amorphous while the others were semi-crystalline. Foreign-body reactions were not observed during the complete follow-up. The SonicWeld Rx and LactoSorb systems reached bone percentages of negative controls after 18 months while the BioSorb Fx and Inion CPS systems reached these levels after 36 months. The SonicWeld Rx system showed the most predictable degradation profile. All the biodegradable systems were safe to use and well-tolerated (i.e., complete implant replacement by bone, no clinical or histological foreign body reactions, no [sterile] abscess formation, no re-interventions needed), but nanoscale residual polymeric fragments were observed at every system's assessment.

## Highlights

• Foreign-body reactions are a major concern of biodegradable osteosyntheses.
• Amorphous poly(DLLA) showed the most predictable degradation profile.
• Nanoscale residual polymeric fragments could still be observed after 4 years.

## Graphical abstract

**Figure:**
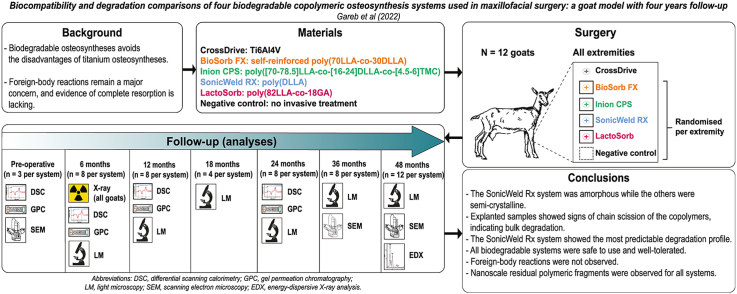

## 1 Introduction

Biodegradable materials, mainly consisting of polymers, are used as temporary implantable medical devices [1]. Biodegradable polymers, such as poly(l-lactic acid) (PLLA), are widely used in different medical disciplines including orthopaedic, trauma and maxillofacial surgery (e.g., in osteosynthesis systems) [[1], [2], [3]], cardiology and thoracic surgery (e.g., in cardiovascular stents) [1], and neurosurgery (e.g., in temporary intracranial pressure, pH and temperature sensors) [1,4]. Since their degradation kinetics and mechanical properties can be easily modulated by using, for example, L- and D-chirality of lactic acid or by copolymerization with different homopolymer ratios, researchers as well as clinicians have been increasingly interested in such biodegradable polymers over the last few decades [1,5,6].

Currently, titanium osteosynthesis systems are considered the gold standard to fixate bone segments in oral and maxillofacial surgery (OMF-surgery) [7,8], and orthopedics and trauma surgery [9]. However, the disadvantages of titanium systems include temperature sensitivity [10], tactile sensations of the plates and screws [11], growth restrictions [12], hampering of imaging and radiotherapy [[13], [14], [15]], presence of titanium particles in lymph nodes [16], extreme stiffness causing stress shielding of the underlying bone [2] and increased risk of medication-related osteonecrosis of the jaws [6,17]. Consequently, titanium systems are removed in a second operation in up to 40% of cases, resulting in accompanying costs and burdens [7,11,18].

The removal rate of polymeric biodegradable osteosynthesis systems in OMF-surgery is less and the disadvantages of titanium osteosyntheses are avoided [7,8]. Biodegradable systems should, preferably, be completely resorbed after 3–12 months [6]. However, sterile abscess formation due to foreign-body reactions remain a major concern, even after >5-years follow-up [7,11,19,20]. Factors that are known to influence foreign-body reactions are implant related (e.g., polymer composition), recipient related (e.g., blood supply), and plate location related (e.g., supraperiosteal versus subperiosteal) [1,21,22].

The most commonly used (co)polymers in biodegradable osteosynthesis systems are PLLA, poly(D,L-lactic acid) (PDLLA), poly(lactic-*co*-glycolic acid) (PLGA), or poly(L-*co*-D,l-lactic acid-*co*-trimethylene carbonate) (P(LLA-*co*-DLLA-*co*-TMC)) [2,7]. These (co)polymers degrade in two phases to eventually form CO_2_ and H_2_O as final products: early degradation via hydrolysis of ester bonds can produce crystalline intermediate products that undergo secondary hydrolysis [5]. Secondary hydrolysis is the rate-limiting step and depends highly on the crystallinity and hydrophobicity of the intermediate products. The reported foreign body reactions (FBR) occur predominately with biodegradable osteosyntheses with a high proportion (i.e., >70%) of PLLA [1,11,[23], [24], [25]] or poly(glycolic acid) (PGA) [1]. More amorphous copolymers such as PDLLA (e.g., 50LLA/50DLA ratio) and PLGA (e.g., 70LLA/30 GA ratio) are more hydrophilic, and degrade and resorb more quickly [26].

Several studies have assessed tissue responses to biodegradable osteosynthesis systems composed of as-polymerized PLLA [23], amorphous PLLA [27], PDLLA [[28], [29], [30]], PLGA [26,29,[31], [32], [33], [34]], and P(LLA-co-DLLA-co-TMC) [35,36], with follow-ups ranging from 6 weeks to 2 years [26,[28], [29], [30], [31], [32], [33], [34],[36], [37], [38]]. Although most of these studies still found residual polymeric particles at the final follow-up, several studies concluded these systems had been resorbed completely by the 1 to 2-year follow-ups [28,29,32]. However, these conclusions were only based on *in vivo* assessments of degradation using light microscopy while the polymeric fragment dimensions which can induce foreign-body reactions can be smaller than the resolution of light microscopy [23]. Furthermore, degradation of these polymers leads to increasing crystallinity and even to the formation of crystalline oligomeric stereo-complexes over time [39,40] that are more stable and resistant to further hydrolytic degradation and resorption than amorphous fragments [1,6,23,41]. Therefore, evidence of complete resorption of polymeric biodegradable systems (i.e., at a nanoscale level) with appropriate follow-up (i.e, >2 years) is still lacking. Moreover, a comparison of biodegradable systems composed of different copolymers in the same model have not been performed and, thus, only indirect comparisons of degradation profiles of biodegradable osteosyntheses are currently available. Hence, there remains a need for an assessment of the degradation of different polymeric biodegradable osteosynthesis systems at nanoscale levels with a long-term follow-up, as this is essential for biocompatibility evaluations as well as to gain knowledge of the development of FBR to such biodegradable polymers.

This study aimed to assess and compare the histological responses (i.e., at macro, micro- and nanoscale levels) of four commonly used copolymeric biodegradable osteosynthesis systems in a goat model from a six-month up to a four-year follow-up. Additionally, the molecular and thermal properties of these systems at different time points were analysed to assess *in vivo* fragmentation and crystallinity, respectively, of the (residual) copolymers with time.

## 2 Materials and methods

This study was conducted following the International Organization for Standardization (ISO) standards [42] and is reported according to the ‘Animal Research: Reporting of In Vivo Experiments’ (ARRIVE) guidelines [43]. The study is fully in agreement with the National Laws and Regulations for Animal Experiments, National Institutes of Health guide for the care and use of Laboratory animals [44], and was approved by the Institutional Animal Care and Use Committee of the University of Groningen (UG)/University Medical Center Groningen (UMCG; DEC 5642A).

### 2.1 Osteosynthesis systems

**Fig. 1:**
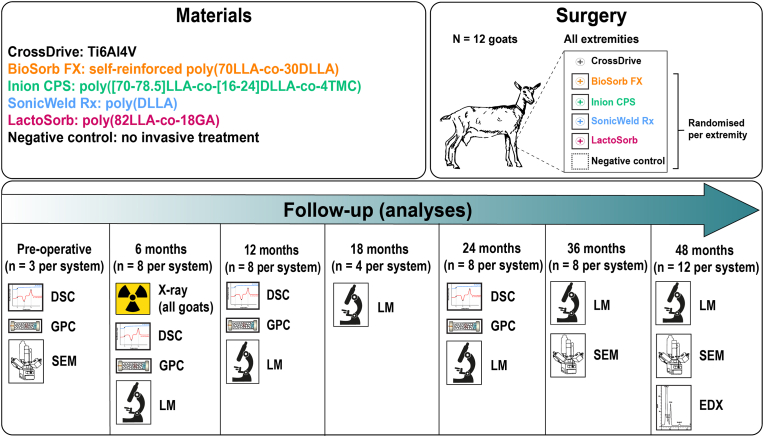
Study design. Note that the study protocol (i.e., assessment at 6, 12, 24, 36, and 48 months [n = 8 per system] with two extra goats [n = 8 per system]) differed from the actual execution as presented here: the goat that died prematurely was assessed at 18 months follow-up (n = 4 per system), one of the extra goats was used to replace the goat that died prematurely, and the other extra goat was sacrificed at the 48-month follow-up (n = 12 per system). *Ti6Al4V, 90/6/4% titanium/aluminum/vanadium; LLA,**l**-lactic acid; DLLA,**d**,**l**-lactic acid; TMC, trimethylene carbonate; GA, glycolic acid; DSC, differential scanning calorimetry; GPC, gel permeation chromatography; LM, light microscopy; SEM, scanning electron microscopy; EDX, Energy-dispersive X-ray analysis.*

Four different copolymeric biodegradable osteosynthesis systems commonly used in OMF-surgery were included [7], viz. BioSorb FX 2.0 × 7 mm (self-reinforced poly(70LLA-*co*-30DLLA) stereo-copolymer; ConMed Linvatec Biomaterials Ltd., Tampere, Finland), Inion CPS 2.0 × 7 mm (poly([70–78.5]LLA-*co*-[16–24]DLLA-*co*-4TMC); Inion Oy, Tampere, Finland), SonicWeld Rx 2.1 × 7 mm (poly(DLLA) stereo-copolymer; KLS Martin Group, Gebrüder Martin GmbH & Co., Tuttlingen, Germany), and LactoSorb 2.0 × 7 mm (poly(82LLA-*co*-18GA) copolymer; Biomet Microfixation, Jacksonville, Florida, USA) [2]. Proton nuclear magnetic resonance analyses (^1^H NMR; Bruker Avance III 400 MHz NMR spectrometer using CDCl_3_ as a solvent at 25 °C) of the materials confirmed that the composition of the polymers was in agreement with the manufacturer's specifications (data not shown). All the systems consisted of a 1-hole plate with a corresponding biodegradable screw or pin [2]. Additionally, a CrossDrive 2.0 × 6 mm screw (90/6/4% titanium/aluminum/vanadium [Ti6Al4V]; KLS Martin Group) were used as a non-degradable reference marker (i.e., to localise the biodegradable implants after complete fragmentation and resorption). As a negative control, an area where no invasive treatment was performed was assessed (Fig. 1). The sizes of plates and screws are given in Supplemental Table S1. The minimal distance between implants was ≥1 cm. All the osteosynthesis systems underwent the manufacturer's sterilization process (i.e., the BioSorb FX, Inion CPS, and SonicWeld Rx systems were sterilized with γ-irradiation at a dose level of 25 kGy and the LactSorb system with two 2-h ethylene oxide (EtO) half cycles with 100% EtO gas at 38 to 43 °C), were implanted before the expiration date, and were applied according to the manufacturer's instructions. Assessment of surface topography of all materials was performed with chromium coating using scanning electron microscopy (SEM; Zeiss Supra55 SEM at 3 kV) [45,46].

### 2.2 Animals and surgical procedure

Twelve skeletally mature female Dutch White goats (16–18 months old and 72–79 kg) were selected (Fig. 1). Skeletally mature goats have similar osseous macro- and microarchitecture, physiology, biomechanical properties, bone composition, and remodelling rates as humans, and are able to generate a FBR to copolymeric biomaterials [47]. Therefore, goats are a suitable and recommended large animal model for preclinical assessment of biomaterials for bone reconstruction [[48], [49], [50], [51], [52]]. Since this was the first study comparing different copolymeric osteosynthesis in a long-term animal model, and since pilot data was not available, sample size calculation was performed using the Fermi approximation method [53]. All systems were implanted in both tibia and radius of each goat. Thus, each goat had a total of 16 biodegradable 1-hole plates with screws/pins (i.e., four of each biodegradable system) and four Ti6Al4V screws implanted (a total of 20 implants per animal). Two goats were planned to be sacrificed at 6, 12, 24, 36 and 48 months follow-up resulting in 8 samples from each biodegradable system per time point. Two extra goats were included for the premature death of a goat so that 8 samples could be studied at each time point. This ensured sufficient samples for reliable histological and molecular analyses [42] while also reducing the number of animals used for scientific research [1,42]. The positions of the biodegradable systems and the negative control were randomised using a computer random number generator by a researcher (LD) who was not involved in the outcome assessment or in the statistical analyses. Prior to surgery, all the goats were acclimatized for two weeks with daily cycles of 12 h light/dark and were fed twice daily with hay and grain. A veterinarian ensured good health by performing complete health assessments. All the goats were housed on a farm specialized in animal research (Overasselt, the Netherlands) and had not been used in previous research.

The surgical procedures were performed by a senior OMF-surgeon (RRMB) and a researcher (NBB) at the Central Animal Laboratory of the UMCG under standard aseptic conditions. General anaesthesia was induced by an intravenous injection of thiopental (15 mg/kg body weight). The goats were intubated and received a stomach probe. Anaesthesia was maintained with a mixture of sevoflurane/30% oxygen through a constant volume ventilator. Vital signs (i.e., heart rate, body temperature, oxygen saturation, and respiration monitoring) were monitored during surgery. The incision sites were disinfected using a diluted betadine solution and saline. Buprenorphine (10 μg/kg body weight) was administered intravenously to reduce postoperative pain. A skin incision was made anterior of the tibia and radius. The skin and underlying soft tissues were transected and reflected from the bone. Using a drilling guide with the titanium screw as a reference, the screw holes were drilled with the prescribed drills (Supplemental Table S1) and tapped with the prescribed taps while cooled with sterile saline. After subperiosteal fixation of osteosynthesis systems, the periosteum and soft tissues were closed tension-free in three layers with Vicryl® 3-0. An additional intramuscular injection of buprenorphine (10 μg/kg body weight) was administered 10 h after the surgical procedure. The goats’ general behaviour, vital signs, wound inflammation, macroscopic swelling, mobility, appetite and defecation were checked daily post-operatively by a veterinarian.

### 2.3 Specimen retrieval and processing

After 6 months, all the animals underwent an X-ray of the surgical sites. The goats were euthanized with an overdose of intravenously injected pentobarbital. Two randomly selected samples of each biodegradable system were retrieved after 6, 12, and 24 months for planned analyses with differential scanning calorimetry (DSC, n = 1) and gel permeation chromatography (GPC, n = 1). However, the DSC analyses of explanted materials could not be performed due to insufficient amounts of remaining material at 6, 12 and 48-months follow-up. The GPC analyses of explanted materials could only be performed of the BioSorb FX system. The remaining amounts of materials of the other three biodegradable systems were also insufficient for GPC analyses. The remaining samples of each biodegradable system were planned to be retrieved for histological processing, i.e., at 6, 12, and 24 months: n = 6 per system; at 36 and 48 months: n = 8 per system. The pH of all the implant sites was measured and noted at every assessment moment.

The histological processing of the samples involved fixating in 4% phosphate-buffered formalin solution for 5 days, decalcifying with 10% aqueous ethylenediaminetetraacetic acid (EDTA) solutions, and dehydration in ascending ethanol concentrations (70–100%). The samples were embedded in poly(glycidyl methacrylate). Poly(methyl methacrylate) was deliberately avoided as it dissolves the polymers of the biodegradable systems and thus would interfere with the study's results (Supplementary Table S2) [54].

Longitudinal histological sections of ∼5 μm thickness were prepared for (polarized) light microscopy (LM) assessment using a rotational microtome (Leica RM 2155) and subsequently stained with hematoxylin and eosin (HE) and Safranin O-fast green (SafO) [54]. The SafO-stained sections were used to verify any occurrence of endochondral ossification. The histological sections were recoded by one researcher (LD) so that both assessors (BG and PB) were blinded.

Histological sections of ∼1 μm thickness were cut for SEM using an ultramicrotome and glass knife, collected on a wet slide, dried and finally sputter-coated with gold. These histological sections were prepared from the ≥36 month follow-up samples without and with observable birefringent fragments. Elemental mapping using energy-dispersive X-ray analysis (EDX) was performed on the samples with birefringent fragments to verify the origin of these fragments. EDX was performed on similar samples but with carbon coating using a X-Max 150 EDX detector (Oxford Instruments) mounted on a Zeiss Supra55 SEM operated at 10 kV, as described previously [45,46].

### 2.4 Differential scanning calorimetry

DSC was performed on the initial samples as well as on the 6, 12, and 24 month follow-up explanted samples. The DSC setup consisted of a PerkinElmer Pyris 1 Differential Scanning Calorimeter (Fremont, CA, USA) and was performed under an inert atmosphere of ultra-high purity N_2_. Indium was used for calibration. The 8–11 mg samples were cooled to 0 °C at 300 °C/min, held for 1 min and then heated to 200 °C at 10 °C/min. The glass transition temperature (T_g_; onset and midpoint), melting temperature (T_m_; onset and midpoint), and melting enthalpy (ΔH_m_) were determined using OriginPro 2019b (OriginLab Corporation, Northampton, MA, USA). The degree of crystallinity was calculated using the following formula [55]:

(1) X_c_ = (ΔH_m_/ΔH*) / Φ_PLLA_* 100%

where X_c_ is the degree of crystallinity (%), ΔH_m_ the determined melting enthalpy of the copolymer (J/g), ΔH* the melting enthalpy of 100% crystalline PLLA (J/g), and Φ_PLLA_ the weight fraction of LLA segments in the copolymer. The melting enthalpy of 100% crystalline PLLA is 93 J/g [56].

### 2.5 Gel permeation chromatography

The weight averaged molecular weight (Mw) and the number averaged molecular weight (Mn), the polydispersity index (PDI), and intrinsic viscosity of the copolymers were determined with GPC. GPC was performed on the initial samples as well as on the 6, 12, and 24 month the explanted samples. The GPC setup consisted of a Viscotek GPCmax VE-2001 GPC solvent/sample module (Malvern, Worcestershire, United Kingdom), a series of ViscoGEL I columns, and a TDA 302 triple detector array consisting of a light scattering detector (i.e., Right Angle Light Scattering and Low Angle Light Scattering), a differential refractive index detector, and a four-capillary differential viscometer. A polystyrene standard (Mn = 64000 g/mol) with a narrow molecular weight distribution was used for calibration. Tetrahydrofuran was used as the eluent.

### 2.6 Histology and histomorphometry

The histological sections were independently assessed by two blinded researchers (BG and PB) using a systematic and validated approach [42]. Each histological section was divided into two pre-defined regions of interest: the supraosseous zone, which is the side of the implant that was not covered by bone at the time of insertion (t = 0) at the periosteal side (i.e., the head of the screw/pin and plate), and the intraosseous zone, which is the side of the implant that was covered by bone at the time of insertion, towards the endosteum (i.e., the shaft of the screw/pin).

Both zones of each histological section were assessed using a semi-quantitative scoring system based on (polarized) light microscopy (Zeiss Axioplan 2, Carl Zeiss Microscopy GmbH) [42,57]. The complete list with scoring-items and their definitions are presented in Supplemental Table S3. Briefly, the scoring items consisted of: implant fragmentation, implant resorption at implant site, type of bone formation, fibrous capsule thickness, necrosis, active remodelling, periosteal or endosteal reaction, birefringent particles in non-implant sites, and the location of any birefringent particles in the non-implant sites. Non-implant site was defined as not being the original site of the implant but was in the same histological section. Additionally, cell responses were scored from the implant and bone interfaces (cells per field at 100× magnification). The scored cells included multinucleated giant cells (MNGCs), polymorphonuclear leukocytes (PMNs), eosinophils, macrophages, lymphocytes, and adipocytes. Likewise, distant cell responses (i.e., not at the implant site but in the same histological section) to birefringence particles were scored. In addition to the above-mentioned cell types, distant osteocytes with birefringence particles were also scored. The percentage of new bone formation per zone, and the total in each histological section, was quantitatively analysed with the aid of the image processing software ImageJ Fiji (version 2.1.0/1.53c) [58]. Any score disagreements between the two blinded researchers was resolved by a discussion.

### 2.7 Statistical analysis

Ordinal data were presented as medians with 25th to 75th percentiles. Nominal data were presented as the number of samples with the corresponding percentage. Continuous data were presented as means ± standard error of the mean (SEM). Univariable statistical comparisons of the ordinal, nominal and continuous data between osteosynthesis systems were performed by applying the Friedman test, Cochran's Q test, or the repeated measure analysis of variance (ANOVA) test, respectively. The inter-rater reliability of the nominal and ordinal data was assessed by calculating the unweighted and quadratic weighted Cohen's kappa, respectively, as well as by calculating the percentage of agreement [59].

Multilevel models were fitted to assess the effect of the different osteosynthesis systems on all the scored items. The fixed effects of the models included the type of osteosynthesis system and follow-up in months. The interaction between the osteosynthesis system and follow-up (system*follow-up) or a quadratic term of follow-up (follow-up [2]) were only included if such a relation was visually observed and if the term improved the model. Model improvement was tested using likelihood-ratio tests. The included random effects were the subjects. All the models yielded an estimated regression coefficient (β) with corresponding 95% confidence intervals (95% CI). In addition, odds ratios were calculated for the nominal and ordinal outcome variables.

P ≤ 0.05 (two-tailed) was considered statistically significant. The Bonferroni correction was applied to all the pairwise comparisons to correct for multiple testing. All the analyses were performed in R, version 4.0.5 of, using the *lme4*-package [60].

## 3 Results

### 3.1 Postoperative care and follow-up

The surgical procedures were performed without any complications. All the osteosynthesis systems were implanted according to the pre-specified protocol. The surgical procedures were well tolerated by all 12 goats. None of the goats showed any deviations in general behaviour, vital signs, appetite and defecation. Mild swelling was noted directly post-operatively around the surgical site, which disappeared without further treatment. During the complete follow-up, no wound inflammation was observed.

At the 18-month follow-up, one goat showed signs of an aching back, most probably due to a previous epileptic seizure, and therefore Carprofen (1.4 mg/kg body weight) was administered subcutaneously. Initially, the goat recovered but after two weeks it showed signs of another epileptic seizure and subsequently died prematurely. An independent veterinarian concluded that the goat's epileptic seizures were not related to the experimental procedures. Specimens were retrieved from this goat and analysed as an 18-month follow-up. No other premature deaths occurred. One of the extra goats was used to replace the goat that died prematurely, the other goat was sacrificed at the 48-month follow-up (samples: at 6, 12, 24 and 36 months: n = 8 per system; at 18 months: n = 4 per system; at 48 months: n = 12 per system; Fig. 1).

### 3.2 Differential scanning calorimetry

The calorimetric properties of the included systems are presented in Table 1. The initial samples showed that the glass transition temperature (midpoint) ranged from 55.3 (Inion CPS plate) to 58.2 °C (BioSorb FX plate). The Inion CPS plate and screw had evident melting peaks at ∼136 °C with ΔH_m1_ of 20–26 J/g, corresponding to a crystallinity of 27.6–40.8% related to PLLA. The LactoSorb screw also showed melting peaks at ∼136 °C with ΔH_m1_ of 14.6 J/g, corresponding to a crystallinity of 19.1%. The melting peaks of the LactoSorb and BioSorb FX plates were minimal. The SonicWeld Rx plate and pin did not show any melting peaks. DSC of the explanted samples from all the systems could not be performed at the 6, 12, and 24 month follow-ups due to insufficient amount remaining material.

**Table 1:** Differential scanning calorimetry results of the initial and explanted materials.

Brand name of system	Part	Copolymer composition	Initial materials						Explanted materials				
									6-, 12-, and 24-months follow-up				
			Tg (onset) (°C)	Tg (midpoint) (°C)	Tm (onset) (°C)	Tm (midpoint) (°C)	ΔHm (J/g)	Xc (%)	Tg (midpoint) (°C)	Tm (onset) (°C)	Tm (midpoint) (°C)	ΔHm (J/g)	Xc (%)
BioSorb FX	Plate	SR poly(70LLA-*co*-30DLLA)	56.6	58.2	–	–	1.87	2.9	Insufficient amounts of remaining material to perform analyses				
	Screw	SR poly(70LLA-*co*-30DLLA)	53.1	55.6	–	–	–	0.0					
Inion CPS	Plate	Poly([70–78.5]LLA-*co*-[16–24]DLLA-*co*-4TMC)	52.4	55.3	128.8	136.4	26.59	36.4 to 40.8					
	Screw	Poly([70–78.5]LLA-*co*-[16–24]DLLA-*co*-4TMC)	53.8	57.2	124.4	136.2	20.12	27.6 to 30.9					
SonicWeld Rx	Plate	Poly(DLLA)	54.8	55.3	–	–	–	0.0					
	Pin	Poly(DLLA)	55.0	55.9	–	–	–	0.0					
LactoSorb	Plate	Poly(82LLA-*co*-18GA)	56.4	57.2	–	–	0.93	1.2					
	Screw	Poly(82LLA-*co*-18GA)	52.0	54.3	125.8	135.5	14.55	19.1					

Abbreviations: Tg, glass transition temperature; C, Celcius; Tm, melting temperature; ΔHm, melting enthalpy; Xc, degree of crystallinity assuming crystallization of PLLA segments only; SR, self-reinforced; LLA, l-lactic acid; DLLA, d,l-lactic acid; TMC, trimethylene carbonate; GA, glycolic acid.

### 3.3 Gel permeation chromatography

The results of the GPC are presented in Table 2. An analysis of the initial samples showed that the Mn ranged from 25.6 (LactoSorb plate) to 64.3 kDa (SonicWeld Rx plate), while the Mw ranged from 62.5 (LactoSorb plate) to 100.2 kDa (SonicWeld Rx plate). The PDI of the initial samples ranged from 1.5 (Inion CPS plate) to 2.5 (LactoSorb screw), indicating substantial differences between systems in the breadths of molecular weight distribution. The intrinsic viscosity ranged from 0.88 (SonicWeld Rx pin) to 1.31 dl/g (BioSorb FX screw). GPC could only be performed on the 6-month follow-up explanted BioSorb FX plate and screw samples as there was insufficient amount of explanted material available from the other systems to perform the analyses. After 6-months, the Mn, Mw, and intrinsic viscosity of the BioSorb plate and screw decreased, while the PDIs of both parts increased.

**Table 2:** Gel permeation chromatography results of the initial and explanted materials.

Brand name of system	Part	Copolymer composition	Initial materials				Explanted materials				
							6-months follow-up				12- and 24-months follow-up
			Mn (kg/mol)	Mw (kg/mol)	Polydispersity index	Intrinsic viscosity (dl/g)	Mn (kg/mol)	Mw (kg/mol)	Polydispersity index	Intrinsic viscosity (dl/g)	
BioSorbFX	Plate	SR poly(70LLA-*co*-30DLLA)	39.9	85.7	2.2	1.14	26.4	61.5	2.3	0.91	Insufficient amounts of remaining material to perform analyses
	Screw	SR poly(70LLA-*co*-30DLLA)	42.3	83.6	2.0	1.31	33.8	77.7	2.3	1.07	
Inion CPS	Plate	Poly([70–78.5]LLA-*co*-[16–24]DLLA-*co*-4TMC)	61.1	92.5	1.5	1.30	Insufficient amounts of remaining material to perform analyses				
	Screw	Poly([70–78.5]LLA-*co*-[16–24]DLLA-*co*-4TMC)	52.4	97.1	1.9	1.15					
SonicWeld Rx	Plate	Poly(DLLA)	64.3	100.2	1.6	1.05					
	Pin	Poly(DLLA)	49.2	81.6	1.7	0.88					
LactoSorb	Plate	Poly(82LLA-*co*-18GA)	25.6	62.5	2.4	0.93					
	Screw	Poly(82LLA-*co*-18GA)	28.0	68.7	2.5	0.96					

Abbreviations: Mn, number averaged molecular weight; Mw, weight averaged molecular weight; kg/mol, 10^3^ g per mol; dl, deciliter; g, gram; SR, self-reinforced; LLA, l-lactic acid; DLLA, d,l-lactic acid; TMC, trimethylene carbonate; GA, glycolic acid.

### 3.4 Surface topography

**Fig. 2:**
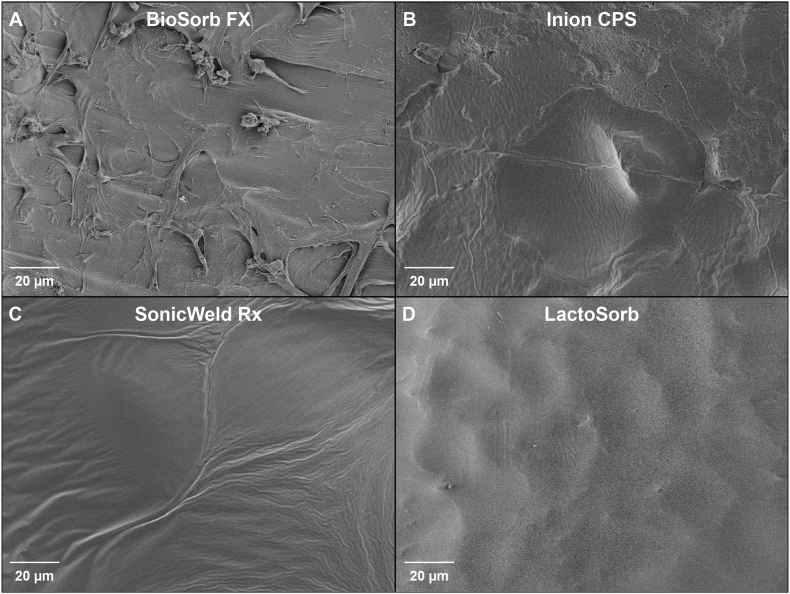
Scanning electron micrographs of the BioSorb FX (A), Inion CPS (B), SonicWeld Rx (C), and LactoSorb (D) surfaces of initial samples at 1500× magnification.

The scanning electron micrographs of the initial samples of the four biodegradable implants are shown in Fig. 2. The BioSorb FX and Inion CPS systems had rough and irregular surfaces while the SonicWeld Rx and LactoSorb systems had smooth and homogenous surfaces. Polymeric fibres could be observed at the surface of the BioSorb FX implant. None of the implants was porous.

### 3.5 X-ray radiographs

The 6-month follow-up X-ray radiographs of the surgical sites (Supplemental Fig. S1) showed that all the osteosynthesis screw holes were still visible. The volume occupied by each osteosynthesis plate was also visible.

### 3.6 Histology and histomorphometry

The inter-rater reliability and percentage of agreement between both assessors of the histological sections ranged from 0.66 to 1.00 and 93.7–100%, respectively (Supplemental Table S4).

**Fig. 3:**
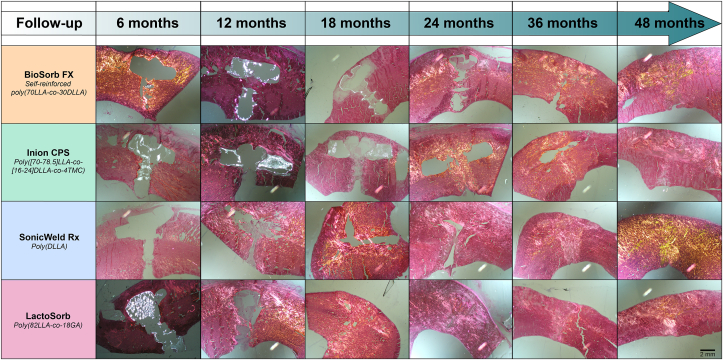
Overview of histological HE-sections assessed with polarized light microscopy (LM-pol) of each osteosynthesis system over time at 12.5× magnification. *HE, hematoxylin and eosin; LLA,**l**-lactic acid; DLLA,**d**,**l**-lactic acid; TMC, trimethylene carbonate; GA, glycolic acid.*

The pH of the implant sites could not be measured as all the implants were overgrown with bone at every assessment moment. An overview of the histological sections assessed with polarized light microscopy (LM-pol) of each osteosynthesis system over time is shown in Fig. 3. At the 6-month follow-up, remnants of the BioSorb FX, Inion CPS and LactoSorb osteosynthesis systems were clearly visible using LM-pol while remnants of the SonicWeld Rx system were not visible. At the 12- and 18-month follow-ups, fragments of the BioSorb FX and Inion CPS systems were still visible but not of the SonicWeld Rx and LactoSorb systems. At the 24-month follow-up, no remnants of any system could be observed at the implant sites using LM-pol. All the implant areas were replaced by bone with time. The overview shows signs of bulk degradation as well as surface erosion of all the included systems (Fig. 3).

**Fig. 4:**
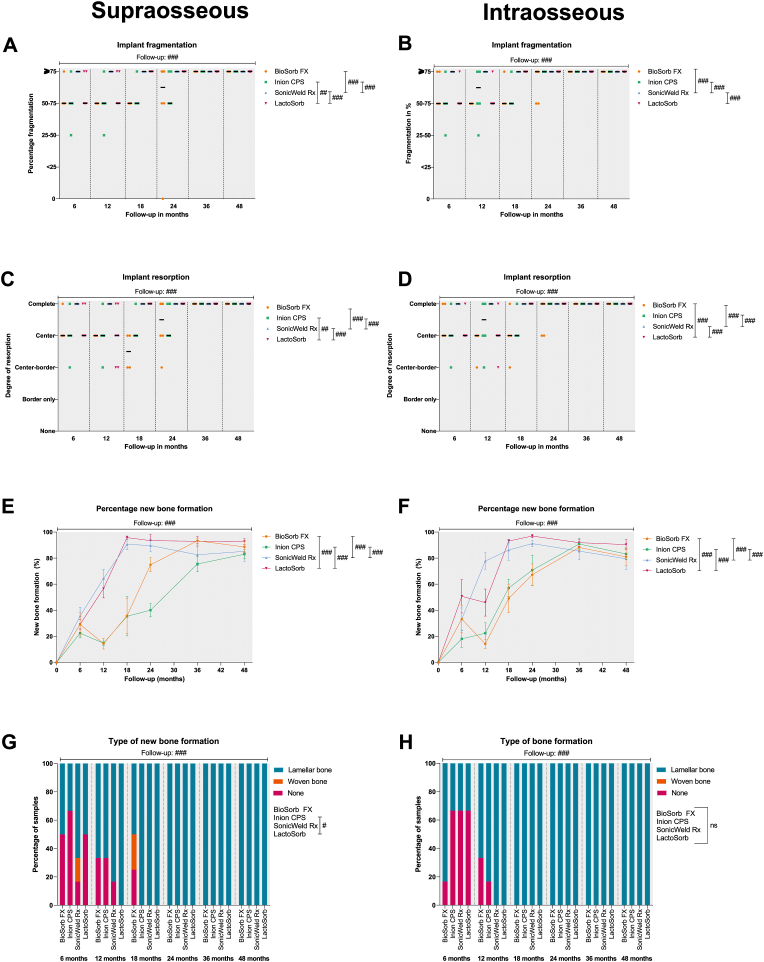
Implant fragmentation at the supraosseous and intraosseous zones (A and B, resp.). Implant resorption at the supraosseous and intraosseous zones (C and D, resp.). Percentage of new bone formation at the supraosseous and intraosseous zones (E and F, resp.). Type of bone formation at the supraosseous and intraosseous zones (G and H, resp.). *Samples: at 6, 12, and* 24 months*: n* = *6 per system; at* 18 months*: n* = *4 per system; at* 36 months*: n* = *8 per system; and at* 48 months*: n* = *12 per system. Black bars represent median values (Fig. A-D). Symbols with error bars represent mean±SEM (Fig. E-F). #, ##, and ### represent P* < *0.05, P* < *0.01, and P* < *0.001, respectively, in the multilevel model analyses including all follow-up data. Ns, non-significant; SEM, standard error of the mean.*

**Fig. 5:**
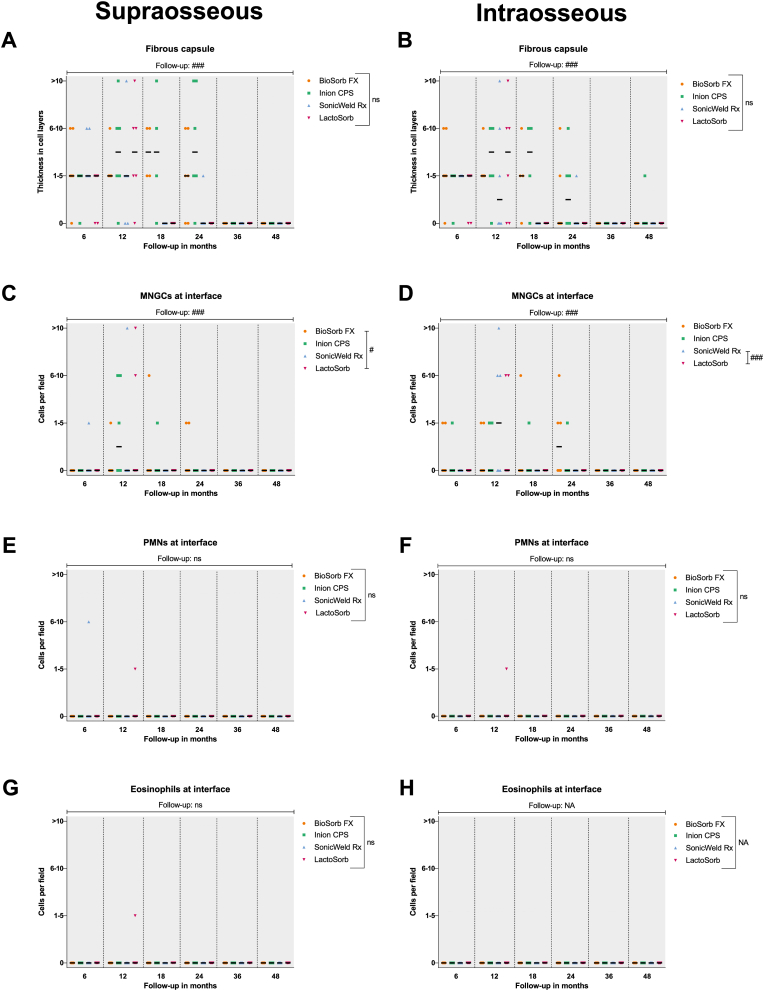
Fibrous capsule at the supraosseous and intraosseous zones (A and B, resp.). Presence of MNGCs at the supraosseous and intraosseous zones with 100× magnification (C and D, resp.). Presence of PMNs at interface at the supraosseous and intraosseous zones with 100× magnification (E and F, resp.). Presence of eosinophils at the supraosseous and intraosseous zones with 100× magnification (G and H, resp.). *Samples: at 6, 12, and* 24 months*: n* = *6 per system; at* 18 months*: n* = *4 per system; at* 36 months*: n* = *8 per system; and at* 48 months*: n* = *12 per system. Black bars represent median values. #, ##, and ### represent P* < *0.05, P* < *0.01, and P* < *0.001, respectively, in the multilevel model analyses including all follow-up data. Ns, non-significant; MNGCs, multinucleated giant cells.*

**Fig. 6:**
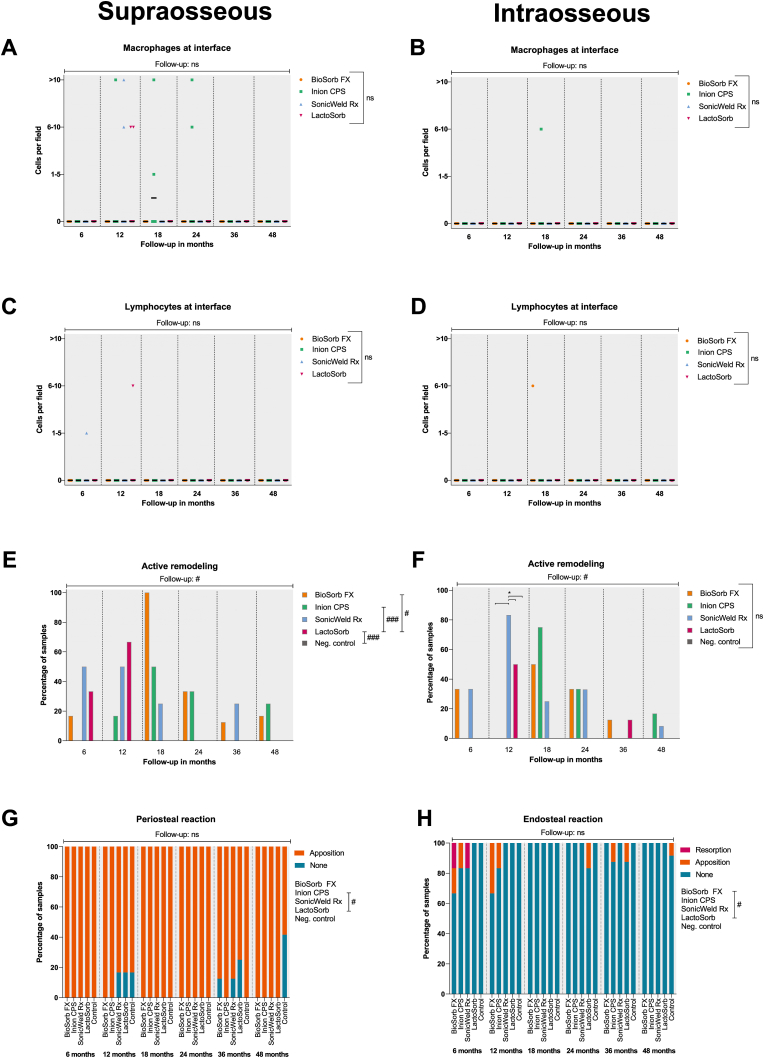
Presence of macrophages at interface at the supraosseous and intraosseous zones with 100× magnification (A and B, resp.). Presence of lymphocytes at the supraosseous and intraosseous zones with 100× magnification (C and D, resp.). Active remodelling at the supraosseous and intraosseous zones (E and F, resp.; the absence of bars represent 0% of samples). Presence of periosteal (G) and endosteal reaction (H). *Samples: at 6, 12, and* 24 months*: n* = *6 per system; at* 18 months*: n* = *4 per system; at* 36 months*: n* = *8 per system; and at* 48 months*: n* = *12 per system. Black bars represent median values (Fig. A-D). #, ##, and ### represent P* < *0.05, P* < *0.01, and P* < *0.001, respectively, in the multilevel model analyses including all follow-up data. Ns, non-significant.*

The scores of all the semi-quantitative scoring items of both zones over the 6–18 month and 24–48 month follow-ups, with univariable analyses at each follow-up point between the different systems, are shown in Supplemental Tables S5 and S6, respectively. Visual presentations of the scoring items are shown in Fig. 3, Fig. 4, Fig. 5, Fig. 6. Adipocytes with birefringent particles at interface (both zones), necrosis (both zones), eosinophils at interface (intraosseous zone), and distant PMNs, eosinophils and lymphocytes with birefringent particles were not observed in any of the histological sections. The multilevel models with estimates, odds ratios (OR) and P-values are presented in Supplemental Tables S7 and S8.

The fragmentation and resorption scores were similar between the supra- and intraosseous zones (Fig. 4A and B and 4C-D, respectively). At the 6-month follow-up, the SonicWeld Rx was completely absent in all the histological sections while the other three systems showed observable implant fragments up to the 24-month follow-up (Fig. 8A and B). The multilevel model of both the supra- and intraosseous zones showed that follow-up (i.e., the effect of time) was significantly associated with fragmentation and resorption of the implant (P < 0.001). On adjusting for the effect of time, the LM(-pol) assessment of the SonicWeld Rx indicated that it was significantly more fragmented and resorbed compared to the other three systems (both P < 0.001; Supplemental Tables S7 and S8).

**Fig. 7:**
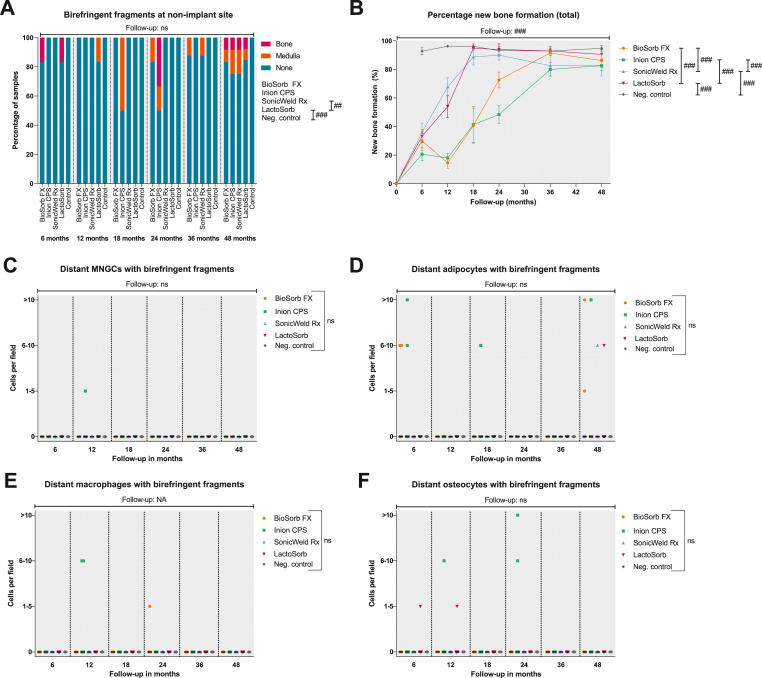
Presence of birefringent fragments at non-implant site (A), percentage of total new bone formation (B), and presence of distant MNGCs (C), distant adipocytes (D), distant macrophages (E), and distant osteocytes (F) with birefringent fragments with 100× magnification. *Samples: at 6, 12, and* 24 months*: n* = *6 per system; at* 18 months*: n* = *4 per system; at* 36 months*: n* = *8 per system; and at* 48 months*: n* = *12 per system. Symbols with error bars represent mean±SEM (Fig. B). Black bars represent median values (Fig. C–F). #, ##, and ### represent P* < *0.05, P* < *0.01, and P* < *0.001, respectively, in the multilevel model analyses including all follow-up data. Ns, non-significant; SEM, standard error of the mean; MNGCs, multinucleated giant cells.*

**Fig. 8:**
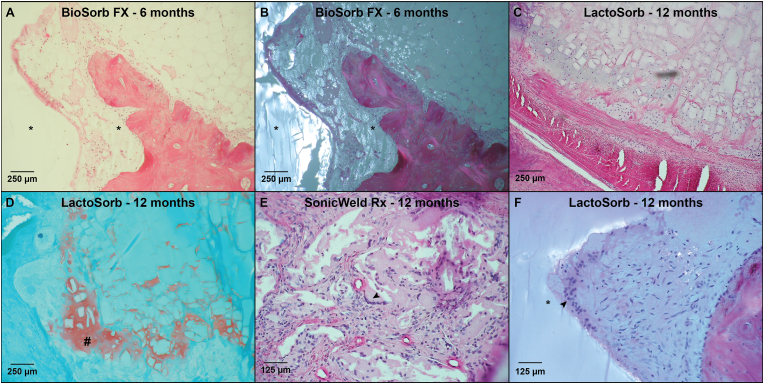
(A & B) HE-sections of the BioSorb FX system at 6-months follow-up under LM and LM-pol, resp., with observable polymer fragments (*). (C & D) HE- and SafO-section of the LactoSorb system at 12-months follow-up, showing the presence of cartilage (D: orange area; #) indicating endochondral ossification. (E) HE-section under LM showing the presence of MNGCs (black arrow) at the supraosseous zone of the SonicWeld Rx system at 12-months follow-up. (F) HE-section under LM-pol showing the presence of MNGCs (black arrow) at the interface of the LactoSorb system (*) at 12-months follow-up. *HE, hematoxylin and eosin; LM, light microscopy; LM-pol, polarized light microscopy; resp., respectively; SafO, Safranin O-fast green, MNGCs, multinucleated giant cells. (For interpretation of the references to colour in this figure legend, the reader is referred to the Web version of this article.)*

The course of new bone formation at the supra- and intraosseous zones of the SonicWeld Rx system and at the supraosseous zone of the LactoSorb system increased constantly up to the 18 month follow-up while the trajectory of new bone formation with the BioSorb FX (both zones), Inion CPS (both zones), and LactoSorb (intraosseous zone) systems showed a deviation at the 12-month follow-up (Fig. 4E and F). The multilevel model showed that the BioSorb FX and Inion CPS systems had significantly lower new bone formation in both zones compared to the SonicWeld Rx and LactoSorb systems (both P = 0.001). In the supraosseous zones, woven bone was observed in the histological section after 6 months (SonicWeld Rx) and 18 months (BioSorb FX) while woven bone was not observed in the intraosseous zones (Fig. 4G and H). The multilevel model demonstrated a significant effect of time (OR 1.11 [1.06; 1.16] per month; P < 0.001) as well as a significant difference in the odds of having new bone formation in the supraosseous zone between the Inion CPS system (OR 0.29 [0.09; 0.99]) and the LactoSorb system (OR 1.0 [reference category]; P = 0.049; Fig. 4G). New lamellar type bone was observed in all the histological sections after 24 months in the supraosseous and after 18 months in the intraosseous zones. The SafO-stained sections confirmed the occurrence of endochondral ossification (Fig. 8C and D). The percentage of total new bone formation (i.e., at the complete implant site) with the SonicWeld Rx and LactoSorb systems reached the same level as the negative control after 18 months while the BioSorb Fx and Inion CPS systems eached similar levels after 36-months (P < 0.001 in favour of SonicWeld Rx and LactoSorb; Table 3; Fig. 7B).

**Table 3:** Multilevel model of percentage new bone formation at the implant site (n = 210).

Model variables	β (95% CI)	P-value
Intercept	24.31 (11.00; 37.60)	**<0.001**
Osteosynthesis system (ref. = LactoSorb)		**<0.001**
Negative control	46.46 (32.45; 60.46)	**<0.001**
BioSorb FX	−32.27 (−46.28;-18.27)	**<0.001**
Inion CPS	−39.60 (−53.61;-25.60)	**<0.001**
SonicWeld Rx	7.50 (−6.51; 21.50)	0.292
Follow-up (months)	3.45 (2.54; 4.37)	**<0.001**
Osteosynthesis system * Follow-up		**<0.001**
Negative control	−1.09 (−1.52;-0.65)	**<0.001**
BioSorb FX	0.58 (0.15; 1.01)	**0.009**
Inion CPS	0.58 (0.14; 1.01)	**0.009**
SonicWeld Rx	−0.37 (−0.80; 0.07)	0.095
Follow-up^2^ (months^2^)	−0.04 (−0.06;-0.03)	**<0.001**

Bold P-values represent statistical significant values. Example: the percentage of new bone formation at the implant site of the BioSorb FX system at 24 month follow-up is estimated to be: 24.31–32.27 + (3.45 × 24) + (0.58 × 24) – (0.04 × 24 × 24) = 65.7%.

Abbreviations: β, estimated coefficient; CI, confidence interval; Ref, reference group.

**Fig. 9:**
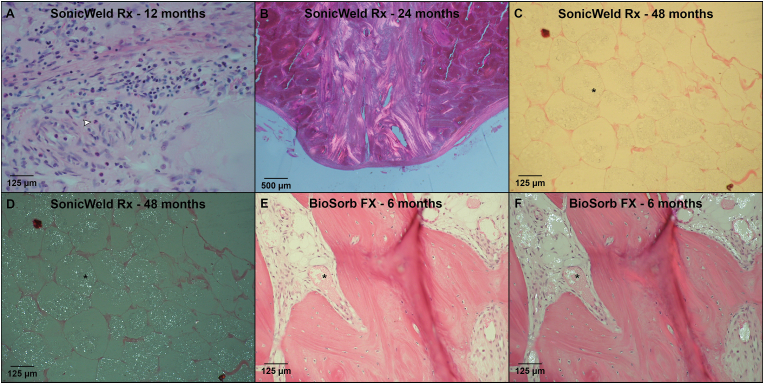
(A) HE-section under LM showing PMNs (white arrow) at the interface of the SonicWeld Rx system at 12-months follow-up. (B) HE-section under LM-pol showing endosteal apposition of the SonicWeld Rx system at 24-months follow-up, in which the distinction between newly formed (light red/purple) and old bone (dark red/purple) is also visible. (C & D) HE-section under LM and LM-pol, resp., of the SonicWeld Rx system at 48-months follow-up with intracellular birefringent fragments (*) in adipocytes at the medulla of bone. (E & F) HE-section under LM and LM-pol, resp., of the BioSorb FX system at 6-months follow-up showing intravascular birefringent fragments (*). *HE, hematoxylin and eosin; LM, light microscopy; LM-pol, polarized light microscopy; resp., respectively; PMNs, polymorphonuclear leukocytes. (For interpretation of the references to colour in this figure legend, the reader is referred to the Web version of this article.)*

The fibrous capsule in the intraosseous zones was generally thinner from the 12- to 24-month follow-ups compared to the supraosseous zones (Fig. 5A and B). In the multilevel model, there was a significant reduction in thickness with time (supraosseous: OR 0.90 [0.87; 0.92] per month; intraosseous: OR 0.90 [0.88; 0.92] per month; both P < 0.001). No significant differences could be detected between the systems when adjusting for follow-up duration (supraosseous: P = 0.232, intraosseous: P = 0.093). The number of MNGCs at the supra- and intraosseous zone interfaces was comparable (Fig. 5C and D). MNGCs were observed in the implant sites of all the systems and were absent from all the sections after the 36-month follow-up (Fig. 8E and F). The multilevel model revealed that increasing follow-up resulted in fewer MNGCs at both zones’ interfaces (both P < 0.001) and that this effect was smaller for the BioSorb FX system (OR 0.96 [0.70; 1.32] per month) at the supraosseous zone compared to the LactoSorb system (0.82 [0.69; 0.98] per month; P = 0.023). The number of PMNs (Fig. 9A), eosinophils, macrophages and lymphocytes at the interface did not differ significantly between all the systems (Fig. 5, Fig. 6D). Remarkably, macrophages were present at the interface of the supraosseous zone in sections of the Inion CPS, SonicWeld Rx, and LactoSorb systems at the 12-, 18-, and 24-month follow-ups (i.e., up to >10 cells per field; Fig. 6A) while macrophages were only observed in a single section of the intraosseous zone of the Inion CPS system at the 18-month follow-up (i.e., 6–10 cells per field) (Fig. 6B). The number of distant MNGCs, macrophages, adipocytes, macrophages and osteocytes with birefringence fragments were not significantly different between the included biodegradable systems (Fig. 7C–F). These distant cells were absent in all negative control sections.

Although the longer follow-up showed lower odds of the presence of active remodelling in both the supra- and intraosseous zones, bone remodelling was still occurring at 48-months in the supraosseous zones of both the BioSorb FX and Inion CPS sections and the intraosseous zones of both the Inion CPS and SonicWeld Rx sections while this was not observed in any of the negative control sections (Fig. 6E and F). The BioSorb FX and Inion CPS sections' intraosseous zone bone remodelling was less compared to that in the LactoSorb sections (Supplemental Table S7). The Inion CPS system resulted in significantly more periosteal apposition compared to the LactoSorb system (OR 2.82 [1.07; 7.40]; P = 0.036). The BioSorb FX, SonicWeld Rx, and the negative control showed similar periosteal reaction. Endosteal reaction was observed in all the systems’ sections from the different follow-ups (Fig. 6, Fig. 9B). The rate at which the endosteal reaction decreased was highest in the BioSorb FX sections (OR 0.91 [0.84; 0.99] per month; P = 0.024). The endosteal reaction of the other three biodegradable systems were comparable with the negative control (Supplemental Table S8).

Birefringent fragments were observed at the implant sites of all the osteosynthesis systems, from 6 to 48 months (Fig. 7A). Most of the birefringent fragments were observed as intracellular accumulation of fragments in adipocytes of the medulla (Fig. 7, Fig. 9C-D; Supplemental Tables S5 and S6). Birefringent fragments were also observed intravascularly after 6-months with the BioSorb FX system (Fig. 9E and F).

### 3.7 SEM and EDX

**Fig. 10:**
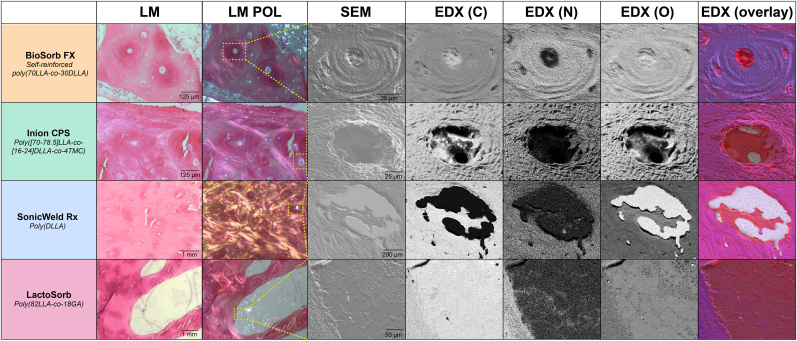
HE-sections (LM and LM-pol), SEM, and EDX by element and with overlay (red: carbon, and blue: nitrogen) of birefringent polymeric residual fragments of every osteosynthesis system at 48-months follow-up. *HE, hematoxylin and eosin; LM, light microscopy; LM-pol, polarized light microscopy; SEM, scanning electron microscopy; EDX, energy-dispersive X-ray analysis; C, carbon; N, nitrogen; O, oxygen. (For interpretation of the references to colour in this figure legend, the reader is referred to the Web version of this article.)*

At the 48-month follow-up, birefringent fragments were observable in all the osteosynthesis systems' LM-pol, SEM, and EDX samples (Fig. 10). The fragments were encapsulated by bone or present in the bone medulla. EDX mapping showed that these birefringent fragments mainly consisted of carbon and oxygen while being nitrogen-free, corresponding with the absence of nitrogen in the implanted materials. Furthermore, birefringent fragments were not observed in any of the control sections. Both aspects substantiated that the crystalline fragments are of polymeric origin (Fig. 10). The 36-month follow-up SEMs also showed typical nanoscale crystalline needle-like structures in the randomly selected vacuoles within the BioSorb FX systems' implant sites’ medulla (Supplemental Fig. S2), without being observable with LM(-pol). None of the other osteosynthesis systems had typical crystalline needle-like structures when analysing locations without birefringent fragments.

## 4 Discussion

Foreign-body reactions to polymeric biodegradable materials remain a major concern in the usage of biomaterials. Currently, comparisons of degradation profiles of different biodegradable copolymers in the same large animal model, as well as evidence of complete resorption of biomaterials, are still lacking. To the best of our knowledge, this is the first study to assess and compare the long-term (i.e., up to 4-years) micro- and nanoscale histological responses to four commonly used copolymeric biodegradable osteosynthesis systems. Additionally, the molecular and thermal properties, and the surface topography of these systems were assessed. A DSC of the initial samples showed that only the SonicWeld Rx plate and pins were completely amorphous while the Inion CPS plate and screws, the LactoSorb screws, and the BioSorb FX plate were clearly semi-crystalline in nature. The differences in PDI and intrinsic viscosity of the initial samples indicated substantial differences between the systems in molecular weight distribution. During the 4-year follow-up, there were no signs of clinical foreign-body reactions and there was no need for re-interventions. Differences in complete implant replacement by bone were observed, viz. the percentage of bone 18 months after implanting the SonicWeld Rx and LactoSorb systems reached the same level as the negative control (i.e., an area where no invasive treatment was performed) while the BioSorb Fx and Inion CPS systems reached similar levels after 36-months. Although all the biodegradable systems were clinically safe to use and were well-tolerated, nanoscale polymeric fragments were observed at every follow-up assessment of all four copolymeric systems, up to 4-years of follow-up.

Biodegradable implants evoke an initial host response after implantation that includes inflammation, proliferation and remodelling of tissue remodelling, and is affected by the degradation products [1]. This host response is mediated by both the innate and adaptive immune systems. Macrophages are the most important innate immune cells during the host response and they play a main role in the outcome of biodegradable implants [1]. The phenotype of macrophages ranges from pro-inflammatory M1 macrophages to anti-inflammatory M2 macrophages [61,62]. After tissue injury, M1 macrophages secrete several inflammatory mediators such as interleukin-1 (IL-1) and tumor necrosis factor-α (TNF-α) to initiate the healing process [1,63]. After the initial inflammatory phase, macrophages switch to a wound-healing phenotype (M2a), secreting growth factors (e.g., platelet-derived growth factor) that promote angiogenesis and cell proliferation [63,64]. Subsequently, macrophages switch to an anti-inflammatory phenotype (M2c) and produce anti-inflammatory cytokines (e.g., IL-10) that leads to the inhibition of the inflammatory response [65].

The adaptive immune system is also involved in the host response to biodegradable implants. Through antigen presentation, macrophages and dendritic cells can activate CD4^+^ T-cells of the adaptive immune system. T helper 1 (T_H_1) cells can induce M1 macrophages by producing interferon-γ and IL-2 [66]. Subsequently, M1 macrophages can produce cytokines and chemokines (e.g., IL-12, CXC-chemokine ligand 9) that intensify the T_H_1 response by recruiting additional T_H_1 cells [1]. In contrast to T_H_1 cells, T_H_2 cells produce anti-inflammatory cytokines (e.g., IL-4 and IL-10) that induce polarization of macrophages towards M2 macrophages. M2 macrophages in turn secrete cytokines (e.g., CC-chemokine ligand 17) that recruits additional T_H_2 cells that tempers the inflammatory response [66]. Imbalances of M1 over M2 macrophages or prominent presence of M1 macrophages may lead to FBR [1]. Therefore, it is essential for a biodegradable implant that a well-controlled and timely switch of M1 to M2 macrophages occurs as this then leads to controlled implant degradation and tissue remodelling, to eventually replace the implant by host tissue (e.g., bone) [1]. Our results show that macrophages at interface were still present up to 24 months follow-up, predominately at the supraosseous zone. The results suggest that the equilibrium between M1 and M2 macrophages at the supraosseous zone is present up to 24 months after implantation, after which macrophage are absent up to 48 months after implantation. On the other hand, apart from one histological section at 18 months follow-up, macrophages at the interface of the intraosseous zone were absent. Together, these results suggest that macrophage activity is particularly located at the supraosseous zone rather than the intraosseous zone in the long-term.

Polymeric biodegradable osteosynthesis systems, including the systems assessed in this study, consist of poly(α-esters) such as PLA, PGA, TMC and their copolymers [1]. Extracellular degradation of poly(α-esters) occur through hydrolysis, enzymatic degradation, and oxidation. During hydrolysis, cleavage of the ester bonds by water results in oligomers and monomers such as lactic acid and glycolic acid [67,68], that can enter the tricarboxylic-acid cycle and are then eliminated as carbon dioxide and water. Furthermore, enzymes secreted by macrophages and derived from blood can contribute to hydrolysis through extracellular hydrolysis [1]. In addition, M1 macrophages can phagocytise biomaterial particles. Inflammatory cells (e.g., macrophages, neutrophils) can induce depolymerisation of polymers by oxidation via the release of reactive oxygen species [69]. Macrophages can also undergo fusion to improve their efficiency and MNGCs [70]. Although the phagocytosis capacity of MNGCs is reduced compared to M1 macrophages, the capacity of extracellular degradation is substantially increased by secreting higher concentrations of enzymes and reactive oxygen species into the interface between the macrophage and implant [70]. In this study, macrophages at the interface were predominately present during 12–24 months follow-up accompanied by the presence of MNGCs at interface. Also, some histological sections showed distant macrophages and MNGCs with intracellular birefringent fragments derived from phagocytized polymeric particles. These results show that MNGCs remain present up to 24 months follow-up, and that phagocytosis of polymeric fragments by inflammatory cells also can take up to 24 months. Future research focussing on degradation and biocompatibility of copolymers included in this study should therefore have a follow-up of ≥24 months so that a proper degradation assessment can be performed.

The progression of the initial host response is affected by the acidic degradation products of poly(α-esters) as they alter the microenvironment in different ways. The lowering of pH intensifies the inflammatory response that results in fibrous encapsulation of the implant [71,72]. Furthermore, the acidic degradation products are autocatalytic resulting in progressive degradation of the remaining polymers and an increase of the inflammatory response. Additionally, bulk degradation leads to fragmentation of the polymer that may result in phagocytized particles within the fibrous tissue [1]. Demineralization of surrounding bone can occur whenever the degradation occurs too quickly and the surrounding tissue fails to eliminate the degradation products [73]. The possibility to induce a FBR (e.g., a sterile abscess formation) is dependent on an equilibrium between the levels of degradation products, the degree of fibrous encapsulation, and the ability of the host to eliminate the degradation products [1]. Short-term FBR are mainly caused by fast-degrading polymers (e.g., PGA) [3] while delayed FBR are often associated with slow-degrading (e.g., PLLA) with high crystallinity and crystalline degradation fragments [23,25,74]. We observed increasing fibrous capsules thicknesses from 6 to 24 months follow-up. Although fibrous encapsulation was present in all system's assessment up to 12 months follow-up, the fibrous capsule remained present at 18 and 24 months follow-up in the BioSorb FX and Inion CPS only. Even though we did not observe a clinical or histological FBR (e.g., sterile abscess formation), these results also emphasize that a follow-up of ≥24 months is essential for assessment of FBR since the degree of fibrous encapsulation is an important factor to induce FBR [1].

Sterile abscess formation due to FBR are presumed to be the main reason for biodegradable plate and screw removal [7,11] and, thus, remain a major concern in the usage of such systems [7,11,23]. Currently, two main hypotheses regarding the aetiology of FBR to these polymeric biomaterials are given. After implantation, the biodegradable polymers are encapsulated by fibrous tissue that acts as a semi-permeable membrane [21]. The first hypothesis is that as the polymer degradation continues over time, the size of the polymeric fragments decreases while the number of particles increases. These particles cannot pass the semi-permeable membrane. Subsequently, the osmotic pressure within the area surrounded by the fibrous layer increases and this results in a clinically observable swelling that, without an intervention, remains *in situ* [11,23]. In this study, no persistent swelling was observed at any implant site during the entire follow-up period. An alternative hypothesis is that, eventually, the acidic polymeric fragments become small enough to pass the membrane. This results in a decrease in pH of the surrounding tissues which then causes an excessive sterile inflammation [75,76] accompanied by phagocytosis of any residual fragments [21]. However, since crystalline fragments are stable and more resistant to further hydrolytic degradation, these residual fragments accumulate in the macrophages and MNGCs, and then remain *in situ*. Besides, extra- and intracellular residual fragments can lead to the accumulation of crystalline oligomeric lactide stereo-complexes over time that are resistant to further hydrolytic degradation [1,39]. These hypotheses could also succeed each other over time.

Our study observed MNGCs at the interface up to the 24-month follow-up. Intracellular accumulation of polymeric fragments was only observed in a few MNGCs in one single histological section of the Inion CPS system. Despite the presence of MNGCs, we did not observe clinical or histological FBR to the implanted copolymeric biomaterials. Previously, MNGCs were characterized as foreign body giant cells and only associated with biomaterial rejection and FBR [77]. Recent research has demonstrated that MNGCs can have both pro-inflammatory and wound-healing aspects [[77], [78], [79]]. MNGCs with a pro-inflammatory character are needed for complete degradation of the polymeric biomaterials while MNGCs with wound-healing aspects are essential for the physiological wound healing process [80]. However, the long-term effect of the presence of MNGCs, and the possibility to induce a FBR, is dependent on a foreign body equilibrium between the presence of the foreign body and presence of MNGCs with degradation capabilities [81]. Whenever intracellular residual polymeric fragments cannot be degraded further, e.g., due to their crystalline nature, this equilibrium is disturbed resulting in an excessive activation of macrophages and subsequent fusion into many more MNGCs than before that may, in turn, lead to bone resorption and/or sterile abscess formation [21,81]. This phenomenon was not observed in our study. In previous research, FBR to poly(70LLA-*co*-30DLLA) was observed in a similar goat model with 12 month follow-up, indicating that the used goat model is capable of generation such a reaction [47]. A possible explanation for not observing FBR in our study could be that all the implants' volumes were too low to induce such a reaction. However, FBR have also been observed in PLLA facial fillers which were of a similar volume as this study's implanted systems [82], so the volume of biomaterial used in this study is not expected to be a limiting factor in inducing FBR.

The DSC-analyses showed that the LactoSorb screw (poly[82LLA-*co*-18GA]) and Inion CPS plate and screws (poly([70–78.5]LLA-*co*-[16–24]DLLA-*co*-4TMC)) were semi-crystalline. According to the manufacturer of the LactoSorb system, the used copolymers have both amorphous and crystalline characteristics [83] which is in line with our analyses. However, the manufacturer of the Inion CPS system states that the copolymers are completely amorphous [84], while the current DSC-analysis indicates a crystallinity of 27–41%. These crystalline regions are more stable and resistant to hydrolytic degradation and resorption than the amorphous regions [23,41]. Furthermore, the amorphous regions degrade faster than the crystalline regions, resulting in inadequate degradation and prolonged presence of crystalline residual fragments, that in turn, may induce FBR [1]. Although this study did not observe FBR, accumulation of residual crystalline fragments was observed up to the 4-year follow-up. Therefore, it is highly preferred to have completely amorphous (co)polymers so that the degradation *in vivo* is predictable [1]. Of the included biodegradable systems, only the SonicWeld Rx (poly[DLLA]) system was completely amorphous and, correspondingly, this system showed the most predictable implant fragmentation and resorption profile as well as new bone formation.

Although it would have been preferable to analyse the explanted samples with DSC and GPC, the limited amount or even lack of explanted materials shows that all four degradable copolymers had been degraded to such an extent that these analyses could not be performed. This was also substantiated by histological assessments at these follow-up moments, that showed advanced fragmentation and resorption in all the histological samples. The Mn, Mw, and intrinsic viscosity of the BioSorb FX plate and screw explanted at 6-months had decreased, indicating chain scission of the copolymer. These findings fit the nature of biodegradable implants with bulk degradation [1].

The birefringent fragments in the 6- and 24-month follow-up non-implant sites may have been from the local distribution of incomplete but ongoing implant fragmentation and resorption. For example, the BioSorb FX and Inion CPS systems had not been completely fragmented and resorbed at the 24-month follow-up (Fig. 4A–D) and birefringent fragments were also observed in the non-implant sites at these assessment moments (Fig. 7A). However, all the systems were absent from the implant sites at the 36- and 48-month follow-ups (Fig. 4A–D) whereas birefringent fragments were increasingly obvious in the non-implant sites. Birefringent fragments were particularly not observed in the non-implant site of the SonicWeld Rx system until the 24-month follow-up but were increasingly present at the 36- and 48-month follow-ups even though implant fragmentation and resorption of this system in the implant site was already completed by the 6-month follow-up (Fig. 4A–D). We observed remarkable accumulations of polymeric birefringent fragments in all the systems’ adipocytes within the medullary bone cavity, even at the 4-year follow-up (Fig. 9C and D). Both the intermediate degradation products of the included copolymers as well as the crystalline oligomeric stereo-complexes that can be formed during degradation over time are hydrophobic [6]. This could explain why these hydrophobic fragments were particularly observed in the adipocytes. It is unlikely that the residual particles were distributed between implant sites, e.g. by circulation or local distribution, since no polymeric fragments were observed in any of the negative control samples despite that the positions of the biodegradable systems and negative control were randomised.

Birefringent fragments derived from as-polymerized PLLA have been observed in previous research with 5-year follow-up [23,27,85], and clinical studies have shown that these PLLA-derived crystalline fragments can induce FBR up to 5.7 years after implantation [23,86]. Since this is the first study that assessed and compared the histological responses of PDLLA, PLGA, and/or P(LLA-*co*-DLLA-*co*-TMC) implants with long-term follow-up, it remains unknown whether the birefringent fragments observed in this study can also induce such a clinical FBR. To the best of our knowledge, the accumulation of polymeric residuals in adipocytes after degradation of PDLLA, PLGA, and/or P(LLA-*co*-DLLA-*co*-TMC) has not been described in the literature before.

Several studies reported complete resorption of the studied systems after 1 to 2-years [29,35,36,87]. However, these conclusions were based on *in vivo* degradation assessments of polymeric fragments using light microscopy whereas polymeric fragment dimensions that induce FBR can be smaller than the light microscopy resolution [23]. This was also shown by our study, viz., fragments were not observable with LM(-pol) but SEM revealed typical nanoscale crystalline needle-like structures in vacuoles from the medulla. Thus, one cannot exclude the presence of residual polymeric fragments if (birefringent) fragments are not observed with light microscopy. Other studies have assessed histological responses to biodegradable osteosyntheses using transmission electronic microscopy (TEM) [23,27]. However, those studies used acetone and/or methyl methacrylate while processing the histological samples. As we have shown in the present study, both dissolve polymeric components of the assessed copolymer biodegradable systems and thus interfere with proper assessment of the polymer in these samples. Therefore, the absence of polymeric residuals in those studies could also be due to the histological specimen processing rather than *in vivo* resorption of these residual fragments.

In addition to polymer composition, the mechanical properties and geometry are also important factors affecting the host reponse [1]. A mismatch between the mechanical properties of an implant can cause micromotions between the implant and host tissue that can lead to FBR [88]. Therefore, it is important that the mechanical properties of biodegradable implants matches with the mechanical properties of the target tissue (e.g., bone). Previous research showed that all assessed systems meet the required mechanical properties for stable fixation of load-sharing maxillofacial fractures and osteotomies [2]. The SonicWeld Rx and BioSorb FX systems showed the most favourable mechanical properties based on tensile, side bending and torsion tests [2]. Since loadings on biodegradable biomaterials can affect degradation and resorption, it is noteworthy that the stress patterns in the tibia and radius are different than that of the maxilla and mandible. However, these differences in stress patterns are particularly important when bridging bone defects (i.e., if the biomaterials are exposed to substantial loads that would otherwise be beared by the bone) [1,89,90]. As we did not create bone defects that had to be bridged by the biomaterials, it is unlikely that stress patterns affected the degradation profile of the assessed biomaterials. Furthermore, studies have shown that geometry and surface topography of the implant also affects the host response. A smooth, well-contoured shape without acute angles induced macrophage polarization towards M2 macrophages (i.e., towards wound repair and an immune regulatory phenotype) whereas implants with acute angles and non-contoured shapes increases the risk of FBR to biomaterials [91,92]. The shape of screws (e.g., of the LactoSorb, BioSorb FX and Inion CPS systems) is by definition different from an ultra-sound welded pin (e.g., from the SonicWeld Rx system). Screws possess acute angles while welded pins are smooth and do not contain acute angles [1]. Furthermore, surface topography analysis showed a smoother surface of the SonicWeld Rx and LactoSorb systems compared to the BioSorb FX and Inion CPS surfaces. Combining both characteristics (i.e., welded pins with smooth surfaces) may explain the favourable degradation profile of the SonicWeld Rx system compared to the other three biodegradable systems [1,93,94]. Since the SonicWeld Rx system is less bulky (i.e., −14% in volume of a 4-hole plate) and the degradation profile is more favourable compared to the BioSorb FX system, we believe the SonicWeld Rx system has the greatest potential as a safe biodegradable copolymeric osteosynthesis system with favourable geometry and mechanical properties for fixation of load-sharing maxillofacial fractures and osteotomies.

Sterilization methods can also alter the physicochemical material properties and may influence biocompatibility [95]. The LactoSorb system was EtO-sterilized while the other three systems were sterilized by γ-irradiation. Although EtO is approved for sterilization of (co)polymeric biomaterials, its usage in recent years has been limited due to its toxicity for humans (i.e., carcinogenic and mutagenic) and environment [95]. An important disadvantage of EtO sterilization is that residues can remain inside the (co)polymer and be leached from the implant *in vivo* up to 3 months after implantation*,* reacting with proteins of the surrounding tissues [95]. Furthermore, EtO can lead to changes in (co)polymers structures (e.g., loss of orientation), molecular weight loss, and increase in crystallization of PLLA that, in turn, affects cell adhesion and proliferation [95]. *In vitro* material comparisons (i.e., inherent viscosity, Tg, stiffness, and energy to break) of EtO-sterilized versus unsterilized LactoSorb systems showed no significant differences [96]. Similar to EtO, γ-irradiation can also result in changes in (co)polymeric properties including cross-linking and/or chain scission. Cross-linking can lead to brittleness, cracking and degradation of the polymer while chain scission results in reduction of molecular weight, decreased pore sizes and rougher surfaces [95]. However, at the irradiation dose needed for sterilization (25 kGy according to ISO standards [97]) these effects are small [95]. In line with previous research [[98], [99], [100], [101], [102], [103], [104], [105]], this study did not show cytotoxicity or other biocompatibility issues during degradation of the four assessed biodegradable systems. Therefore, the effects of both approved and validated sterilization methods are considered not to interfere with the biocompatibility comparisons between the assessed systems.

Based on the results of this study, several key factors can be assigned for a predictable degradation profile. It is preferred to (1) have completely amorphous (co)polymers (e.g., poly[DLLA]), (2) low intrinsic viscosity, (3) a well-contoured shape without acute angles (e.g., by welding pins instead of using screws), (4) a smooth and homogenous surface, and (5) low implant volume but with sufficient mechanical properties for the purpose of the implant [2]. The histological assessment showed no substantial differences between the supra- and intraosseous zones of all included systems, indicating that the degradation of both zones are uniform and is a less important factor for predictable degradation.

Large animal models are the gold standard to assess long-term host response to (biodegradable) implanted devices [1,42] since skeletally mature goats have similar osseous macro- and microarchitecture, physiology, biomechanical properties, bone composition, and remodelling rates as humans, and are able to generate a FBR to copolymeric biomaterials [47]. The model used simulates the *in vivo* degradation and host response of the assessed biomaterials in humans as closely as possible [1,52]. The host response reported in this study can therefore also be expected to be present in humans after implantation of the assessed biodegradable copolymeric osteosynthesis systems [1,42,52]. However, it is important to note that this study only assessed subperiosteally implanted biodegradable materials while in some medical disciplines biomaterials are placed supraperiosteally (e.g., in orthopaedic surgery). Supraperiosteally placed biodegradable implants tend to degrade and resorb slower compared to subperiosteally placed implants [106]. Therefore, translation of these results to copolymeric biodegradable materials that are implanted supraperiosteally should be done with caution.

One of the strengths of our work is that this is the first study to perform histological assessments (i.e., with LM, LM-pol, SEM, and EDX) of four different biodegradable copolymers with a long-term follow-up of a large animal model. Furthermore, all the sections were independently assessed by two blinded researchers using a systematic and validated approach, with good to excellent inter-rater reliability. Additionally, molecular and thermal properties were assessed at baseline, as well as of the explanted osteosynthesis systems, while X-ray radiographs were also taken at the 6-month follow-up. Finally, the animal model was optimized by carrying out extremity-level implantations enabling intra-animal comparisons at individual time-points and during follow-up, thereby increasing the reliability while reducing the number of animals used for the research.

Limitations of this study include not being able to measure the pH because all the implants were overgrown with bone. We did not try to access the implants by drilling holes in the overgrown bone to perform pH measurements because of the possibility of damaging the implant which could, in turn, interfere with the (histological) samples. Furthermore, as polymeric fragments were still present in all the implant systems’ 4-year follow-up sections, extending the follow-up beyond 4-years would be of high scientific value. However, in The Netherlands, a 4-year follow-up is the maximum allowed period for animal research.

## 5 Conclusions

The present study showed no signs of FBR to the BioSorb FX, Inion CPS, SonicWeld Rx, and LactoSorb biodegradable copolymeric osteosynthesis systems during a 4-year follow-up period. The SonicWeld Rx system showed the most predictable profile of implant fragmentation and resorption as well as new bone formation at the implant site. Although all the biodegradable systems were clinically safe to use and were well-tolerated (i.e., complete implant replacement by bone, no clinical or histological FBR, no [sterile] abscess formation, no re-interventions needed), nanoscale polymeric fragments of all four copolymeric systems were observed at every follow-up assessment up to the 4-year follow-up. These residual fragments had predominately accumulated in the adipocytes in the medullary cavity of bone. Whether these nanoparticles may be harmful on the long run (i.e., >4 years) is not clear.

## CRediT authorship contribution statement

**Barzi Gareb:** Methodology, Investigation, Data curation, Project administration, Formal analysis, Visualization, Writing – original draft. **Nico B. van Bakelen:** Conceptualization, Methodology, Investigation, Data curation, Project administration, Writing – review & editing. **Léon Driessen:** Conceptualization, Methodology, Validation, Investigation, Data curation, Resources, Writing – review & editing. **Pieter Buma:** Conceptualization, Methodology, Investigation, Data curation, Project administration, Supervision, Writing – review & editing. **Jeroen Kuipers:** Validation, Investigation, Supervision, Writing – review & editing. **Dirk W. Grijpma:** Conceptualization, Methodology, Validation, Resources, Writing – review & editing. **Arjan Vissink:** Resources, Supervision, Writing – review & editing. **Ruud R.M. Bos:** Conceptualization, Methodology, Supervision, Investigation, Data curation, Project administration, Resources, Funding acquisition, Supervision, Writing – review & editing. **Baucke van Minnen:** Resources, Supervision, Funding acquisition, Writing – review & editing.

## Declaration of competing interest

None.
