# Supplementary material for: Biocompatibility and degradation comparisons of four biodegradable copolymeric osteosynthesis systems used in maxillofacial surgery: A goat model with four years follow-up
Source: Bioact Mater. 2022 Jan 19;17:439–56. doi: 10.1016/j.bioactmat.2022.01.015 (PMC8961280; doi:10.1016/j.bioactmat.2022.01.015)
Supplement: Multimedia component 1 [file mmc1.docx]

**Supplemental Files**

**Biocompatibility and degradation comparisons of four biodegradable copolymeric osteosynthesis systems used in maxillofacial surgery: a goat model with four years follow-up**

Barzi Gareb*^1^, Nico B. van Bakelen^1^, Léon Driessen^2^, Pieter Buma^2^, Jeroen Kuipers^3^, Dirk W. Grijpma^4^, Arjan Vissink^1^, Ruud R.M. Bos^1^, Baucke van Minnen^1^

^1^University of Groningen, University Medical Center Groningen, Department of Oral and Maxillofacial Surgery, Hanzeplein 1, 9713 GZ Groningen, The Netherlands, P.O. Box 30001, 9700 RB Groningen, The Netherlands.

^2^Radboud University Medical Center, Radboud Institute for Molecular Life Sciences, Orthopaedic Research Lab, Nijmegen, The Netherlands.

^3^University of Groningen, University Medical Center Groningen, Department of Biomedical Sciences of Cells and Systems, Groningen, The Netherlands

^4^University of Twente, TechMed Centre, Department of Biomaterials Science and Technology, Enschede, Netherlands

**Corresponding author (*)**

Barzi Gareb

P.O. Box 30001, 9700 RB Groningen, The Netherlands

Telephone number: +31 503611054

Fax number: +31 503612831

E-mail address: b.gareb@umcg.nl

**Supplemental Tables**

**Supplemental Table S1.** Specifications of the included osteosynthesis systems.

**Supplemental Table S2.** Solubility tests of the included osteosynthesis systems in different mediums used for histological processing.

**Supplemental Table S3.** Items scored in the assessment of the histological sections.

**Supplemental Table S4.** Inter-rater reliability and percentage of agreement between both assessors of histological sections.

**Supplemental Table S5.** Scores of all semi-quantitative scoring items of both zones after 6 to 18 months.

**Supplemental Table S6.** Scores of all semi-quantitative scoring items of both zones after 24 to 48 months.

**Supplemental Table S7.** Multilevel models of each outcome scoring item at the supraosseous zone.

**Supplemental Table S8.** Multilevel models of each outcome scoring item at the intraosseous zone and at non-implant site.

**Supplemental Figures**

**Supplemental Figure S1.** X-ray radiographs of the surgical sites at 6-months follow-up.

**Supplemental Figure S2.** Scanning electron microscopy image of crystalline, needle-like structures of nanoscale in randomly selected vacuoles of the medulla after implantation of the BioSorb FX system at 36 months follow-up (magnification 52.860x).

**Supplemental Table S1.** Specifications of the included osteosynthesis systems.

| **Brand name** | **Manufacturer** | **Plate composition** | **Screw/pin composition** | **Drill diameter (mm)** | **Tap diameter (mm)** | **Screw/pin diameter (mm)** | **Screw/pin length (mm)** | **Plate length (mm)** | **Plate width (mm)** | **Plate thickness (mm)** |
| --- | --- | --- | --- | --- | --- | --- | --- | --- | --- | --- |
| ***Biodegradable osteosynthesis systems*** | | | | | | | | | | |
| BioSorb FX 2.0mm | ConMed Linvatec Biomaterials Ltd. (Tampere, Finland) | SR poly(70LLA-co-30DLLA) | SR poly(70LLA-co-30DLLA) | 1.5 | 2.0 | 2.0 | 7.0 | 6.3 | 5.5 | 1.3 |
| Inion CPS 2.0mm | Inion Oy (Tampere, Finland) | Poly([70-78.5]LLA-co-[16-24]DLLA-co-4TMC)^1^ | Poly([70-78.5]LLA-co-[16-24]DLLA-co-4TMC)^1^ | 1.75 | 2.0 | 2.0 | 7.0 | 7.0 | 7.0 | 1.3 |
| SonicWeld Rx 2.1mm | KLS Martin Group (Gebrüder Martin GmbH & Co., Tuttlingen, Germany) | Poly(DLLA) | Poly(DLLA) | 1.6 | None | 2.1 | 7.0 | 6.5 | 6.0 | 1.0 |
| LactoSorb 2.0mm | Biomet Microfixation (Jacksonville, Florida) | Poly(82LLA-co-18GA) | Poly(82LLA-co-18GA) | 1.7 | 2.0 | 2.0 | 7.0 | 7.1 | 7.0 | 1.3 |
| ***Non-degradable reference marker*** | | | | | | | | | | |
| CrossDrive 2.0mm (2006) | KLS Martin Group (Gebrüder Martin GmbH & Co., Tuttlingen, Germany) | NA | 90% titanium  6% aluminium  4%vanadium (Ti6Al4V) | 1.5 | None | 2.0 | 6.0 | NA | | |

SR: self-reinforced; PLLA,  poly-L-lactic acid; PDLLA, poly-D,L-lactic acid; TMC, trimethylene carbonate; PGA, poly-glycolic acid, NA, not applicable.

^1^The manufacturer does not publicly report the exact composition of the copolymers;

**Supplemental Table S2.** Solubility tests of the included osteosynthesis systems in different mediums used for histological processing.

|  |  |  | **Plate thickness after 24-hours of complete submersion in the specific medium** | | | | | | | | | |
| --- | --- | --- | --- | --- | --- | --- | --- | --- | --- | --- | --- | --- |
| **Osteosynthesis system** | **Polymer composition** | **Initial plate thickness** | **Formalin** | **Ethanol 70%** | **Ethanol 96%** | **Ethanol 100%** | **Xylene** | **Acetone** | **MMA** | **GMA** | **EDTA** | **Formic acid** |
| BioSorb FX | SR poly(70LLA-co-30DLLA) | 1.3 mm | 1.3 mm | 1.3 mm | 1.3 mm | 1.3 mm | 1.5 mm | Dissolved | Dissolved | 1.3 mm | 1.3 mm | 1.3 mm |
| Inion CPS | Poly([70-78.5]LLA-co-[16-24]DLLA-co-4TMC)^1^ | 1.3 mm | 1.3 mm | 1.3 mm | 1.3 mm | 1.3 mm | 1.3 mm | Dissolved | Deformed | 1.3 mm | 1.3 mm | 1.3 mm |
| SonicWeld Rx | Poly(DLLA) | 1.0 mm | 1.0 mm | 1.0 mm | 1.0 mm | 1.0 mm | Dissolved | Dissolved | Dissolved | 1.0 mm | 1.0 mm | 1.0 mm |
| LactoSorb | Poly(82LLA-co-18GA) | 1.3 mm | 1.3 mm | 1.3 mm | 1.3 mm | 1.3 mm | 1.3 mm | Deformed | Dissolved | 1.3 mm | 1.3 mm | 1.3 mm |

Abbreviations: MMA, methyl methacrylate; GMA, glycidyl methacrylate; EDTA, Ethylenediaminetetraacetic acid.

^1^The manufacturer does not publicly report the exact composition of the copolymers;

**Supplemental Table S3.** Items scored in the assessment of the histological sections.

| **Scoring-item** | **Sections scored** | **Possible scores** |
| --- | --- | --- |
| 1. Fragmentation of implant | Supra- and intraosseous | 0: 0%  1: <25%  2: 25-50%  3: 50-75%  4: ≥75% |
| 2. Resorption of implant | Supra- and intraosseous | 0: none (i.e., implant completely visible)  1: border only  2: center-border  3: center  4: complete (i.e. implant not visible with light microscopy) |
| 3. Percentage new bone formation | Supra- and intraosseous, and total | Quantitatively analysed with the aid of image processing software (ImageJ Fiji). |
| 4. Type of new bone formation | Supra- and intraosseous | None, woven or lamellar bone. |
| 5. Fibrous capsule thickness | Supra- and intraosseous | 0: 0 cell layers thickness  1: 1-5 cell layers thickness  2: 6-10 cell layers thickness  3: >10 cell layers thickness |
| 6. Cells in interface at 100x magnification, scored per cell type | Supra- and intraosseous | 0: 0 cells per field  1: 1-5 cells per field  2: 6-10 cells per field  3: >10 cells per field |
| 1. Foreign-body giant cells |  |  |
| 1. Polymorphonuclear leukocytes |  |  |
| 1. Eosinophils |  |  |
| 1. Adipocytes with birefringent particles |  |  |
| 1. Macrophages |  |  |
| 1. Lymphocytes |  |  |
| 7. Distant cells with birefringent particles at 100x magnification, scored per cell type | Total | 0: 0 cells per field  1: 1-5 cells per field  2: 6-10 cells per field  3: >10 cells per field |
| 1. Foreign-body giant cells |  |  |
| 1. Polymorphonuclear leukocytes |  |  |
| 1. Eosinophils |  |  |
| 1. Adipocytes |  |  |
| 1. Macrophages |  |  |
| 1. Lymphocytes |  |  |
| 1. Osteocytes |  |  |
| 8. Necrosis | Supra- and intraosseous | Yes or no.  *Yes, if one of these criteria were observed::*   - *Increased eosinophilia* - *Glassy homogenous appearance* - *‘Moth-eaten’ appearance of cytoplasm* - *Dystrophic calcifications* - *Presence of ‘mylein figures’ (i.e., whorled phospholipid masses)* - *Karyolysis* - *Pyknosis* - *Karyorrhexis* |
| 9. Active remodelling | Supra- and intraosseous | Yes or no.  *Defined as: osteoclasts and osteoblasts present and active at the same surface.* |
| 10. Endosteal reaction | Intraosseous | Yes or no. |
| 11. Periosteal reaction | Supraosseous | Yes or no. |
| 12. Birefringent material at non-implant site |  | *Visible using polarized light microscopy.* |
| 1. Present | Supra- and intraosseous | Yes or no |
| 1. Location |  | Description of location. |
| 1. Type of cells |  | Description of cell types. |

**Supplemental Table S4.** Inter-rater reliability and percentage of agreement between both assessors of histological sections.

| **Scoring item**  **(number of possible categories)** | **Percentage of agreement** | **Cohen’s kappa^a^** |
| --- | --- | --- |
| 1. Fragmentation (4) | 99.1 | 0.98 |
| 2. Resorption (4) | 98.5 | 0.96 |
| 3. Type of new bone formation (3) | 98.5 | 0.92 |
| 4. Fibrous capsule thickness (4) | 93.7 | 0.87 |
| 5. Cells in interface (4) |  |  |
| 1. Foreign-body giant cells | 96.7 | 0.94 |
| 1. Polymorphonuclear leukocytes | 97.6 | 0.95 |
| 1. Eosinophils | 97.9 | 0.95 |
| 1. Adipocytes with birefringent particles | 98.2 | 0.95 |
| 1. Macrophages | 97.3 | 0.94 |
| 1. Lymphocytes | 97.6 | 0.94 |
| 6. Distant cells with birefringent particles (4) |  |  |
| 1. Foreign-body giant cells | 100 | 1.00 |
| 1. Polymorphonuclear leukocytes | 100 | 1.00 |
| 1. Eosinophils | 100 | 1.00 |
| 1. Adipocytes | 98.2 | 0.84 |
| 1. Macrophages | 100 | 1.00 |
| 1. Lymphocytes | 100 | 1.00 |
| 1. Osteocytes | 99.4 | 0.89 |
| 7. Necrosis (2) | 100 | NA^b^ |
| 8. Active remodelling (2) | 97.0 | 0.82 |
| 9. Endosteal reaction (2) | 97.0 | 0.77 |
| 10. Periosteal reaction (2) | 98.2 | 0.66 |
| 11. Birefringent material at non-implant site |  |  |
| 1. Present (2) | 98.5 | 0.97 |
| 1. Location (3) | 98.5 | 0.87 |
| 1. Type of cells (3) | 98.8 | 0.90 |

NA: not applicable. ^a^For categorical scoring items, the unweighted Cohen’s kappa was calculated while for scoring items with ordinal categories the quadratic weighted Cohen’s kappa was calculated. ^b^Both observers did not observe necrosis in any of the samples, thus no Cohen’s kappa could be calculated.

**Supplemental Table S5.** Scores of all semi-quantitative scoring items of both zones after 6 to 18 months.

|  | **6 months** | | | | | | **12 months** | | | | | | **18 months** | | | | | |
| --- | --- | --- | --- | --- | --- | --- | --- | --- | --- | --- | --- | --- | --- | --- | --- | --- | --- | --- |
|  | **BioSorbFX** | **Inion CPS** | **SonicWeld Rx** | **LactoSorb** | **Negative control** | **P-value** | **BioSorbFX** | **Inion CPS** | **SonicWeld Rx** | **LactoSorb** | **Negative control** | **P-value** | **BioSorbFX** | **Inion CPS** | **SonicWeld Rx** | **LactoSorb** | **Negative control** | **P-value** |
| **Scoring item** | N=6 | N=6 | N=6 | N=6 | N=6 |  | N=6 | N=6 | N=6 | N=6 | N=6 |  | N=4 | N=4 | N=4 | N=4 | N=4 |  |
| **Fragmentation score*** |  |  |  |  |  |  |  |  |  |  |  |  |  |  |  |  |  |  |
| **Supraosseous** | 3 (3-3.25) | 3 (2.75-3.25) | 4 (4-4) | 3 (3-4) | NA | **0.028** | 3 (3-3) | 3 (2.75-3.25) | 4 (4-4) | 3 (3-4) | NA | **0.010** | 3 (3-3) | 3 (3-3.75) | 4 (4-4) | 4 (4-4) | NA | **0.017** |
| **Intraosseous** | 3 (3-4) | 3 (2.75-3.25) | 4 (4-4) | 3 (3-3.25) | NA | **0.017** | 3 (3-3) | 3.5 (2.75-4) | 4 (4-4) | 3 (3-3.25) | NA | **0.015** | 3 (3-3.75) | 3 (3-3.75) | 4 (4-4) | 4 (4-4) | NA | 0.052 |
| **Resorption score*** |  |  |  |  |  |  |  |  |  |  |  |  |  |  |  |  |  |  |
| **Supraosseous** | 3 (3-3.25) | 3 (2.75-3.25) | 4 (4-4) | 3 (3-4) | NA | **0.028** | 3 (3-3) | 3 (2.75-3.25) | 4 (4-4) | 3 (2-4) | NA | **0.026** | 2.5 (2-3) | 3 (3-3.75) | 4 (4-4) | 4 (4-4) | NA | **0.016** |
| **Intraosseous** | 3 (3-4) | 3 (2.75-3.25) | 4 (4-4) | 3 (3-3.25) | NA | **0.017** | 3 (2.75-3) | 3.5 (2.75-4) | 4 (4-4) | 3 (2.75-3.25) | NA | **0.015** | 3 (2.25-3.75) | 3 (3-3.75) | 4 (4-4) | 4 (4-4) | NA | 0.056 |
| **New bone formation^#^, %** |  |  |  |  |  |  |  |  |  |  |  |  |  |  |  |  |  |  |
| **Supraosseous** | 29.1±8.8 | 22.4±3.6 | 35.6±6.6 | 29.2±3.7 | NA | 0.592 | 14.4±4.2 | 14.7±1.6^a^ | 64.4±6.9^a^ | 56.7±7.3 | NA | **0.004** | 35.8±13.6 | 35.3±15.1 | 90.6±4.1 | 95.8±1.2 | NA | **0.016** |
| **Intraosseous** | 33.2±10.4 | 18.1± 6.6 | 33.3± 8.4 | 39.0±10.5 |  | 0.546 | 14.1±3.4^b^ | 22.3±8.0 | 77.7±6.3^b^ | 45.9 ±10.4 |  | **<0.001** | 49.1±11.0 | 57.1±6.7 | 86.2±7.8 | 93.1±1.5 |  | **0.036** |
| **Total** | 30.0±6.7^c^ | 20.6±4.4^d^ | 35.9±6.5^e^ | 33.2±4.4^f^ | 92.7±2.4^c-f^ | **<0.001** | 14.5±3.8^g,h^ | 18.0±2.9^i,j^ | 67.7±6.4^g,i^ | 54.1±7.9^k^ | 96.3±0.9^h,j,k^ | **<0.001** | 40.5±12.4 | 41.4±12.7 | 88.7±5.7 | 94.8±1.3 | 96.2±1.7 | **0.001** |
| **Type of new bone formation head, n (%)** |  |  |  |  |  |  |  |  |  |  |  |  |  |  |  |  |  |  |
| **Supraosseous** |  |  |  |  |  | 0.533 |  |  |  |  |  | 0.695 |  |  |  |  |  | 0.200 |
| **None** | 3 (50) | 4 (50) | 1 (17) | 3 (50) | NA |  | 2 (33) | 2 (33) | 1 (17) | - | NA |  | 1 (25) | - | - | - | NA |  |
| **Woven** | - | - | 1 (17) | - |  |  | - | - | - | - |  |  | 1 (25) | - | - | - |  |  |
| **Lamellar** | 3 (50) | 2 (33) | 4 (67) | 3 (50) |  |  | 4 (67) | 4 (67) | 5 (83) | 6 (100) |  |  | 2 (50) | 4 (100) | 4 (100) | 4 (100) |  |  |
| **Intraosseous** |  |  |  |  |  | 0.310 |  |  |  |  |  | 0.573 |  |  |  |  |  | NA |
| **None** | 1 (17) | 4 (67) | 4 (67) | 4 (67) | NA |  | 2 (33) | 1 (17) | - | - | NA |  | - | - | - | - | NA |  |
| **Woven** | - | - | - | - |  |  | - | - | - | - |  |  | - | - | - | - |  |  |
| **Lamellar** | 5 (83) | 2 (33) | 2 (33) | 2 (33) |  |  | 4 (67) | 5 (83) | 6 (100) | 6 (100) |  |  | 4 (100) | 4 (100) | 4 (100) | 4 (100) |  |  |
| **Thickness of fibrous capsule, score^*^** |  |  |  |  |  |  |  |  |  |  |  |  |  |  |  |  |  |  |
| **Supraosseous** | 1 (0.75-2) | 1 (0.75-1) | 1 (1-2) | 1 (0-1) | NA | 0.153 | 1 (1-1.25) | 1.5 (0.75-2.25) | 1 (0-1.5) | 1.5 (0.75-2.25) | NA | 0.418 | 1.5 (1-2) | 1.5 (0.25-2.75) | 0 (0-0) | 0 (0-0) | NA | **0.023** |
| **Intraosseous** | 1 (0.75-1) | 1 (0.75-1) | 1 (1-1) | 1 (0-1) |  | 0.290 | 1 (1-1) | 1 (0.75-1) | 0.5 (0-1) | 1 (0-1) |  | 0.861 | 1 (0.25-1) | 1 (0.25-1) | 0 (0-0) | 0 (0-0) |  | 0.052 |
| **MNGCs at interface, score*** |  |  |  |  |  |  |  |  |  |  |  |  |  |  |  |  |  |  |
| **Supraosseous** | 0 (0-0) | 0 (0-0) | 0 (0-0.25) | 0 (0-0) | NA | 0.392 | 0 (0-0.25) | 0.5 (0-2) | 0 (0-0.75) | 0 (0-2.25) | NA | 0.334 | 0 (0-1.5) | 0 (0-0.75) | 0 (0-0) | 0 (0-0) | NA | 0.392 |
| **Intraosseous** | 0 (0-1) | 0 (0-0.25) | 0 (0-0) | 0 (0-0) |  | 0.194 | 0 (0-1) | 0 (0-1) | 1 (0-2.25) | 0 (0-2) |  | 0.448 | 0 (0-1.5) | 0 (0-0.75) | 0 (0-0) | 0 (0-0) |  | 0.392 |
| **PMNs at interface, score*** |  |  |  |  |  |  |  |  |  |  |  |  |  |  |  |  |  |  |
| **Supraosseous** | 0 (0-0) | 0 (0-0) | 0 (0-0.5) | 0 (0-0) | NA | 0.392 | 0 (0-0) | 0 (0-0) | 0 (0-0) | 0 (0-0.25) | NA | 0.392 | 0 (0-0) | 0 (0-0) | 0 (0-0) | 0 (0-0) | NA | >0.99 |
| **Intraosseous** | 0 (0-0) | 0 (0-0) | 0 (0-0) | 0 (0-0) |  | >0.99 | 0 (0-0) | 0 (0-0) | 0 (0-0) | 0 (0-0.25) |  | 0.392 | 0 (0-0) | 0 (0-0) | 0 (0-0) | 0 (0-0) |  | >0.99 |
| **Eosinophils at interface, score*** |  |  |  |  |  |  |  |  |  |  |  |  |  |  |  |  |  |  |
| **Supraosseous** | 0 (0-0) | 0 (0-0) | 0 (0-0) | 0 (0-0) | NA | >0.99 | 0 (0-0) | 0 (0-0) | 0 (0-0) | 0 (0-0.25) | NA | 0.392 | 0 (0-0) | 0 (0-0) | 0 (0-0) | 0 (0-0) | NA | >0.99 |
| **Intraosseous** | 0 (0-0) | 0 (0-0) | 0 (0-0) | 0 (0-0) |  | >0.99 | 0 (0-0) | 0 (0-0) | 0 (0-0) | 0 (0-0) |  | >0.99 | 0 (0-0) | 0 (0-0) | 0 (0-0) | 0 (0-0) |  | >0.99 |
| **Adipocytes with birefringent particles at interface, score*** |  |  |  |  |  |  |  |  |  |  |  |  |  |  |  |  |  |  |
| **Supraosseous** | 0 (0-0) | 0 (0-0) | 0 (0-0) | 0 (0-0) | NA | >0.99 | 0 (0-0) | 0 (0-0) | 0 (0-0) | 0 (0-0) | NA | >0.99 | 0 (0-0) | 0 (0-0) | 0 (0-0) | 0 (0-0) | NA | >0.99 |
| **Intraosseous** | 0 (0-0) | 0 (0-0) | 0 (0-0) | 0 (0-0) |  | >0.99 | 0 (0-0) | 0 (0-0) | 0 (0-0) | 0 (0-0) |  | >0.99 | 0 (0-0) | 0 (0-0) | 0 (0-0) | 0 (0-0) |  | >0.99 |
| **Macrophages at interface, score*** |  |  |  |  |  |  |  |  |  |  |  |  |  |  |  |  |  |  |
| **Supraosseous** | 0 (0-0) | 0 (0-0) | 0 (0-0) | 0 (0-0) | NA | >0.99 | 0 (0-0) | 0 (0-0.75) | 0 (0-2.25) | 0 (0-2) | NA | 0.498 | 0 (0-0) | 0.5 (0-2.5) | 0 (0-0) | 0 (0-0) | NA | 0.112 |
| **Intraosseous** | 0 (0-0) | 0 (0-0) | 0 (0-0) | 0 (0-0) |  | >0.99 | 0 (0-0) | 0 (0-0) | 0 (0-0) | 0 (0-0) |  | >0.99 | 0 (0-0) | 0 (0-1.5) | 0 (0-0) | 0 (0-0) |  | 0.392 |
| **Lymphocytes at interface, score*** |  |  |  |  |  |  |  |  |  |  |  |  |  |  |  |  |  |  |
| **Supraosseous** | 0 (0-0) | 0 (0-0) | 0 (0-0.25) | 0 (0-0) | NA | 0.392 | 0 (0-0) | 0 (0-0) | 0 (0-0) | 0 (0-0.5) | NA | 0.392 | 0 (0-0) | 0 (0-0) | 0 (0-0) | 0 (0-0) | NA | >0.99 |
| **Stem** | 0 (0-0) | 0 (0-0) | 0 (0-0) | 0 (0-0) |  | >0.99 | 0 (0-0) | 0 (0-0) | 0 (0-0) | 0 (0-0) |  | >0.99 | 0 (0-1.5) | 0 (0-0) | 0 (0-0) | 0 (0-0) |  | 0.392 |
| **Distant cells with birefringent particles** |  |  |  |  |  |  |  |  |  |  |  |  |  |  |  |  |  |  |
| **MNGCs, score*** | 0 (0-0) | 0 (0-0) | 0 (0-0) | 0 (0-0) | 0 (0-0) | >0.99 | 0 (0-0) | 0 (0-0.25) | 0 (0-0) | 0 (0-0) | 0 (0-0) | 0.392 | 0 (0-0) | 0 (0-0) | 0 (0-0) | 0 (0-0) | 0 (0-0) | >0.99 |
| **PMNs, score*** | 0 (0-0) | 0 (0-0) | 0 (0-0) | 0 (0-0) | 0 (0-0) | >0.99 | 0 (0-0) | 0 (0-0) | 0 (0-0) | 0 (0-0) | 0 (0-0) | >0.99 | 0 (0-0) | 0 (0-0) | 0 (0-0) | 0 (0-0) | 0 (0-0) | >0.99 |
| **Eosinophils, score*** | 0 (0-0) | 0 (0-0) | 0 (0-0) | 0 (0-0) | 0 (0-0) | >0.99 | 0 (0-0) | 0 (0-0) | 0 (0-0) | 0 (0-0) | 0 (0-0) | >0.99 | 0 (0-0) | 0 (0-0) | 0 (0-0) | 0 (0-0) | 0 (0-0) | >0.99 |
| **Adipocytes, score*** | 0 (0-2) | 0 (0-2.25) | 0 (0-0) | 0 (0-0) | 0 (0-0) | 0.187 | 0 (0-0) | 0 (0-0) | 0 (0-0) | 0 (0-0) | 0 (0-0) | >0.99 | 0 (0-0) | 0 (0-1.5) | 0 (0-0) | 0 (0-0) | 0 (0-0) | 0.392 |
| **Macrophages, score*** | 0 (0-0) | 0 (0-0) | 0 (0-0) | 0 (0-0) | 0 (0-0) | >0.99 | 0 (0-0) | 0 (0-2) | 0 (0-0) | 0 (0-0) | 0 (0-0) | 0.112 | 0 (0-0) | 0 (0-0) | 0 (0-0) | 0 (0-0) | 0 (0-0) | >0.99 |
| **Lymphocytes, score*** | 0 (0-0) | 0 (0-0) | 0 (0-0) | 0 (0-0) | 0 (0-0) | >0.99 | 0 (0-0) | 0 (0-0) | 0 (0-0) | 0 (0-0) | 0 (0-0) | >0.99 | 0 (0-0) | 0 (0-0) | 0 (0-0) | 0 (0-0) | 0 (0-0) | >0.99 |
| **Osteocytes, score*** | 0 (0-0) | 0 (0-0) | 0 (0-0) | 0 (0-0.25) | 0 (0-0) | 0.392 | 0 (0-0) | 0 (0-0.5) | 0 (0-0) | 0 (0-0.25) | 0 (0-0) | 0.527 | 0 (0-0) | 0 (0-0) | 0 (0-0) | 0 (0-0) | 0 (0-0) | >0.99 |
| **Necrosis, n (yes, %)** |  |  |  |  |  |  |  |  |  |  |  |  |  |  |  |  |  |  |
| **Supraosseous** | - | - | - | - | - | NA | - | - | - | - | - | NA | - | - | - | - | - | NA |
| **Intraosseous** | - | - | - | - | - | NA | - | - | - | - | - | NA | - | - | - | - | - | NA |
| **Active remodelling, n (yes, %)** |  |  |  |  |  |  |  |  |  |  |  |  |  |  |  |  |  |  |
| **Supraosseous** | 1 (17) | - | 3 (50) | 2 (33) | - | 0.075 | - | 1 (17) | 3 (50) | 4 (67) | - | **0.038** | 4 (100) | 2 (50) | 1 (25) | - | - | **0.037** |
| **Intraosseous** | 2 (33) | - | 2 (303) | - | - | 0.144 | -^l^ | -^m^ | 5 (83)^l,m,n^ | 4 (67) | -^n^ | **0.002** | 2 (50) | 3 (75) | 1 (25) | 2 (33) | - | 0.147 |
| **Periosteal reaction, n (%)** |  |  |  |  |  | NA |  |  |  |  |  | >0.99 |  |  |  |  |  | NA |
| **None** | - | - | - | - | - |  | - | - | 1 (17) | 1 (17) | 1 (17) |  | - | - | - | - | - |  |
| **Apposition** | 6 (100) | 6 (100) | 6 (100) | 6 (100) | 6 (100) |  | 6 (100) | 6 (100) | 5 (83) | 5 (83) | 5 (83) |  | 4 (100) | 4 (100) | 4 (100) | 4 (100) | 4 (100) |  |
| **Resorption** | - | - | - | - | - |  | - | - | - | - | - |  | - | - | - | - | - |  |
| **Endosteal reaction, n (%)** |  |  |  |  |  |  |  |  |  |  |  |  |  |  |  |  |  |  |
| **None** | 4 (67) | 5 (83) | 5 (83) | 6 (100) | 6 (100) |  | 4 (67) | 5 (83) | 6 (100) | 6 (100) | 6 (100) |  | 4 (100) | 4 (100) | 4 (100) | 4 (100) | 4 (100) |  |
| **Apposition** | 1 (17) | 1 (17) | - | - | - |  | 2 (33) | 1 (17) | - | - | - |  | - | - | - | - | - |  |
| **Resorption** | 1 (17) | - | 1 (17) | - | - |  | - | - | - | - | - |  | - | - | - | - | - |  |
| **Birefringent particles at non-implant site, n (yes, %)** | 1 (17) | - | - | 1 (17) | - | 0.558 | - | - | - | 1 (17) | - | 0.406 | - | 2 (50) | - | - | - | 0.092 |
| **Location** | Bone and intravascular | NA | NA | Bone | NA |  | NA | NA | NA | Medulla | NA |  | NA | Medulla | NA | NA | NA |  |
| **Type of cells** | Adipocytes | NA | NA | Osteocyte | NA |  | NA | NA | NA | Adipocytes | NA |  | NA | Adipocytes | NA | NA | NA |  |

^*^ Data presented as median (25^th^-75^th^ percentile). ^#^Data presented as mean ± SEM. Pairwise comparison with Bonferroni correction for multiple testing: ^a^P=0.012, ^b^P=0.019, ^c^P=0.003, ^d^P<0.001, ^e^P=0.001, ^f^P=0.001, ^g^P=0.016, ^h^P<0.001, ^i^P=0.013, ^j^P<0.001, ^k^P=0.037, ^l^P=0.039, ^m^P=0.039, and ^n^P=0.039. Bold P-values are statistically significant (i.e. P<0.05). MNGCs, multinucleated giant cells; PMNs, polymorphonuclear leukocytes ; -: none (0.0%); NA: not applicable; SEM: standard error of the mean.

**Supplemental Table S6.** Scores of all semi-quantitative scoring items of both zones after 24 to 48 months.

|  | **24 months** | | | | | | **36 months** | | | | | | **48 months** | | | | | |
| --- | --- | --- | --- | --- | --- | --- | --- | --- | --- | --- | --- | --- | --- | --- | --- | --- | --- | --- |
|  | **BioSorbFX** | **Inion CPS** | **SonicWeld Rx** | **LactoSorb** | **Negative control** | **P-value** | **BioSorbFX** | **Inion CPS** | **SonicWeld Rx** | **LactoSorb** | **Negative control** | **P-value** | **BioSorbFX** | **Inion CPS** | **SonicWeld Rx** | **LactoSorb** | **Negative control** | **P-value** |
| **Scoring item** | N=6 | N=6 | N=6 | N=6 | N=6 |  | N=8 | N=8 | N=8 | N=8 | N=8 |  | N=12 | N=12 | N=12 | N=12 | N=12 |  |
| **Fragmentation score*** |  |  |  |  |  |  |  |  |  |  |  |  |  |  |  |  |  |  |
| **Supraosseous** | 3.5 (2.25-4) | 3 (3-4) | 4 (4-4) | 4 (4-4) | NA | **0.029** | 4 (4-4) | 4 (4-4) | 4 (4-4) | 4 (4-4) | NA | >0.99 | 4 (4-4) | 4 (4-4) | 4 (4-4) | 4 (4-4) | NA | >0.99 |
| **Intraosseous** | 4 (3-4) | 4 (4-4) | 4 (4-4) | 4 (4-4) | NA | 0.112 | 4 (4-4) | 4 (4-4) | 4 (4-4) | 4 (4-4) | NA | >0.99 | 4 (4-4) | 4 (4-4) | 4 (4-4) | 4 (4-4) | NA | >0.99 |
| **Resorption score*** |  |  |  |  |  |  |  |  |  |  |  |  |  |  |  |  |  |  |
| **Supraosseous** | 3.5 (2.75-4) | 3 (3-4) | 4 (4-4) | 4 (4-4) | NA | **0.029** | 4 (4-4) | 4 (4-4) | 4 (4-4) | 4 (4-4) | NA | >0.99 | 4 (4-4) | 4 (4-4) | 4 (4-4) | 4 (4-4) | NA | >0.99 |
| **Intraosseous** | 4 (3-4) | 4 (4-4) | 4 (4-4) | 4 (4-4) | NA | 0.112 | 4 (4-4) | 4 (4-4) | 4 (4-4) | 4 (4-4) | NA | >0.99 | 4 (4-4) | 4 (4-4) | 4 (4-4) | 4 (4-4) | NA | >0.99 |
| **New bone formation^#^, %** |  |  |  |  |  |  |  |  |  |  |  |  |  |  |  |  |  |  |
| **Supraosseous** | 74.9±5.4^a^ | 40.1±5.1^a,b,c^ | 89.6±4.4^b^ | 93.4±4.6^c^ | NA | **<0.001** | 93.3±2.0 | 75.5±5.9 | 82.6±8.3 | 92.9±3.6 | NA | 0.123 | 88.6±4.6 | 83.0±3.5 | 85.2±7.6 | 92.7±2.1 | NA | 0.517 |
| **Intraosseous** | 67.2±8.3^d^ | 70.7±11.5 | 91.1±2.4 | 97.0±1.3^d^ |  | **0.043** | 88.2±2.4 | 90.9±4.2 | 85.4±6.4 | 91.9±2.7 |  | 0.724 | 81.0±6.6 | 83.2±4.2 | 79.5±8.3 | 90.4±3.8 |  | 0.606 |
| **Total** | 72.6±5.6 | 48.3±6.1^e,f,g^ | 90.0±3.4^e^ | 93.9±3.9^f^ | 93.1±2.6^g^ | **<0.001** | 91.5±1.7 | 80.0±4.7 | 82.6±7.2 | 92.9±3.0 | 92.9±2.0 | 0.075 | 86.1±4.9 | 82.7±3.3 | 83.0±7.6 | 90.5±3.3 | 94.8±1.9 | 0.339 |
| **Type of new bone formation head, n (%)** |  |  |  |  |  |  |  |  |  |  |  |  |  |  |  |  |  |  |
| **Supraosseous** |  |  |  |  |  | NA |  |  |  |  |  | NA |  |  |  |  |  | NA |
| **None** | - | - | - | - | NA |  | - | - | - | - | NA |  | - | - | - | - | NA |  |
| **Woven** | - | - | - | - |  |  | - | - | - | - |  |  | - | - | - | - |  |  |
| **Lamellar** | 6 (100) | 6 (100) | 6 (100) | 6 (100) |  |  | 8 (100) | 8 (100) | 8 (100) | 8 (100) |  |  | 12 (100) | 12 (100) | 12 (100) | 12 (100) |  |  |
| **Intraosseous** |  |  |  |  |  | NA |  |  |  |  |  | NA |  |  |  |  |  | NA |
| **None** | - | - | - | - | NA |  | - | - | - | - | NA |  |  | - | - | - | NA |  |
| **Woven** | - | - | - | - |  |  | - | - | - | - |  |  |  | - | - | - |  |  |
| **Lamellar** | 6 (100) | 6 (100) | 6 (100) | 6 (100) |  |  | 8 (100) | 8 (100) | 8 (100) | 8 (100) |  |  | 12 (100) | 12 (100) | 12 (100) | 12 (100) |  |  |
| **Thickness of fibrous capsule, score^*^** |  |  |  |  |  |  |  |  |  |  |  |  |  |  |  |  |  |  |
| **Supraosseous** | 1 (0-2) | 1.5 (0.75-3) | 0 (0-0.25) | 0 (0-0) | NA | **0.029** | 0 (0-0) | 0 (0-0) | 0 (0-0) | 0 (0-0) | NA | >0.99 | 0 (0-0) | 0 (0-0) | 0 (0-0) | 0 (0-0) | NA | >0.99 |
| **Intraosseous** | 0 (0-1.25) | 0.5 (0-1.25) | 0 (0-0.25) | 0 (0-0) |  | 0.232 | 0 (0-0) | 0 (0-0) | 0 (0-0) | 0 (0-0) |  | >0.99 | 0 (0-0) | 0 (0-0) | 0 (0-0) | 0 (0-0) |  | 0.392 |
| **MNGCs at interface, score*** |  |  |  |  |  |  |  |  |  |  |  |  |  |  |  |  |  |  |
| **Supraosseous** | 0 (0-1) | 0 (0-0) | 0 (0-0) | 0 (0-0) | NA | 0.112 | 0 (0-0) | 0 (0-0) | 0 (0-0) | 0 (0-0) | NA | >0.99 | 0 (0-0) | 0 (0-0) | 0 (0-0) | 0 (0-0) | NA | >0.99 |
| **Intraosseous** | 0.5 (0-1.25) | 0 (0-0.25) | 0 (0-0) | 0 (0-0) |  | **0.043** | 0 (0-0) | 0 (0-0) | 0 (0-0) | 0 (0-0) |  | >0.99 | 0 (0-0) | 0 (0-0) | 0 (0-0) | 0 (0-0) |  | >0.99 |
| **PMNs at interface, score*** |  |  |  |  |  |  |  |  |  |  |  |  |  |  |  |  |  |  |
| **Supraosseous** | 0 (0-0) | 0 (0-0) | 0 (0-0) | 0 (0-0) | NA | >0.99 | 0 (0-0) | 0 (0-0) | 0 (0-0) | 0 (0-0) | NA | >0.99 | 0 (0-0) | 0 (0-0) | 0 (0-0) | 0 (0-0) | NA | >0.99 |
| **Intraosseous** | 0 (0-0) | 0 (0-0) | 0 (0-0) | 0 (0-0) |  | >0.99 | 0 (0-0) | 0 (0-0) | 0 (0-0) | 0 (0-0) |  | >0.99 | 0 (0-0) | 0 (0-0) | 0 (0-0) | 0 (0-0) |  | >0.99 |
| **Eosinophils at interface, score*** |  |  |  |  |  |  |  |  |  |  |  |  |  |  |  |  |  |  |
| **Supraosseous** | 0 (0-0) | 0 (0-0) | 0 (0-0) | 0 (0-0) | NA | >0.99 | 0 (0-0) | 0 (0-0) | 0 (0-0) | 0 (0-0) | NA | >0.99 | 0 (0-0) | 0 (0-0) | 0 (0-0) | 0 (0-0) | NA | >0.99 |
| **Intraosseous** | 0 (0-0) | 0 (0-0) | 0 (0-0) | 0 (0-0) |  | >0.99 | 0 (0-0) | 0 (0-0) | 0 (0-0) | 0 (0-0) |  | >0.99 | 0 (0-0) | 0 (0-0) | 0 (0-0) | 0 (0-0) |  | >0.99 |
| **Adipocytes with birefringent particles at interface, score*** |  |  |  |  |  |  |  |  |  |  |  |  |  |  |  |  |  |  |
| **Supraosseous** | 0 (0-0) | 0 (0-0) | 0 (0-0) | 0 (0-0) | NA | >0.99 | 0 (0-0) | 0 (0-0) | 0 (0-0) | 0 (0-0) | NA | >0.99 | 0 (0-0) | 0 (0-0) | 0 (0-0) | 0 (0-0) | NA | >0.99 |
| **Intraosseous** | 0 (0-0) | 0 (0-0) | 0 (0-0) | 0 (0-0) |  | >0.99 | 0 (0-0) | 0 (0-0) | 0 (0-0) | 0 (0-0) |  | >0.99 | 0 (0-0) | 0 (0-0) | 0 (0-0) | 0 (0-0) |  | >0.99 |
| **Macrophages at interface, score*** |  |  |  |  |  |  |  |  |  |  |  |  |  |  |  |  |  |  |
| **Supraosseous** | 0 (0-0) | 0 (0-2.25) | 0 (0-0) | 0 (0-0) | NA | 0.112 | 0 (0-0) | 0 (0-0) | 0 (0-0) | 0 (0-0) | NA | >0.99 | 0 (0-0) | 0 (0-0) | 0 (0-0) | 0 (0-0) | NA | >0.99 |
| **Intraosseous** | 0 (0-0) | 0 (0-0) | 0 (0-0) | 0 (0-0) |  | >0.99 | 0 (0-0) | 0 (0-0) | 0 (0-0) | 0 (0-0) |  | >0.99 | 0 (0-0) | 0 (0-0) | 0 (0-0) | 0 (0-0) |  | >0.99 |
| **Lymphocytes at interface, score*** |  |  |  |  |  |  |  |  |  |  |  |  |  |  |  |  |  |  |
| **Supraosseous** | 0 (0-0) | 0 (0-0) | 0 (0-0) | 0 (0-0) | NA | >0.99 | 0 (0-0) | 0 (0-0) | 0 (0-0) | 0 (0-0) | NA | >0.99 | 0 (0-0) | 0 (0-0) | 0 (0-0) | 0 (0-0) | NA | >0.99 |
| **Intraosseous** | 0 (0-0) | 0 (0-0) | 0 (0-0) | 0 (0-0) |  | >0.99 | 0 (0-0) | 0 (0-0) | 0 (0-0) | 0 (0-0) |  | >0.99 | 0 (0-0) | 0 (0-0) | 0 (0-0) | 0 (0-0) |  | >0.99 |
| **Distant cells with birefringent particles** |  |  |  |  |  |  |  |  |  |  |  |  |  |  |  |  |  |  |
| **MNGCs, score*** | 0 (0-0) | 0 (0-0) | 0 (0-0) | 0 (0-0) | 0 (0-0) | >0.99 | 0 (0-0) | 0 (0-0) | 0 (0-0) | 0 (0-0) | 0 (0-0) | >0.99 | 0 (0-0) | 0 (0-0) | 0 (0-0) | 0 (0-0) | 0 (0-0) | >0.99 |
| **PMNs, score*** | 0 (0-0) | 0 (0-0) | 0 (0-0) | 0 (0-0) | 0 (0-0) | >0.99 | 0 (0-0) | 0 (0-0) | 0 (0-0) | 0 (0-0) | 0 (0-0) | >0.99 | 0 (0-0) | 0 (0-0) | 0 (0-0) | 0 (0-0) | 0 (0-0) | >0.99 |
| **Eosinophils, score*** | 0 (0-0) | 0 (0-0) | 0 (0-0) | 0 (0-0) | 0 (0-0) | >0.99 | 0 (0-0) | 0 (0-0) | 0 (0-0) | 0 (0-0) | 0 (0-0) | >0.99 | 0 (0-0) | 0 (0-0) | 0 (0-0) | 0 (0-0) | 0 (0-0) | >0.99 |
| **Adipocytes, score*** | 0 (0-0) | 0 (0-0) | 0 (0-0) | 0 (0-0) | 0 (0-0) | >0.99 | 0 (0-0) | 0 (0-0) | 0 (0-0) | 0 (0-0) | 0 (0-0) | >0.99 | 0 (0-0) | 0 (0-0) | 0 (0-0) | 0 (0-0) | 0 (0-0) | 0.896 |
| **Macrophages, score*** | 0 (0-0.25) | 0 (0-0) | 0 (0-0) | 0 (0-0) | 0 (0-0) | 0.392 | 0 (0-0) | 0 (0-0) | 0 (0-0) | 0 (0-0) | 0 (0-0) | >0.99 | 0 (0-0) | 0 (0-0) | 0 (0-0) | 0 (0-0) | 0 (0-0) | >0.99 |
| **Lymphocytes, score*** | 0 (0-0) | 0 (0-0) | 0 (0-0) | 0 (0-0) | 0 (0-0) | >0.99 | 0 (0-0) | 0 (0-0) | 0 (0-0) | 0 (0-0) | 0 (0-0) | >0.99 | 0 (0-0) | 0 (0-0) | 0 (0-0) | 0 (0-0) | 0 (0-0) | >0.99 |
| **Osteocytes, score*** | 0 (0-0) | 0 (0-2.25) | 0 (0-0) | 0 (0-0) | 0 (0-0) | 0.112 | 0 (0-0) | 0 (0-0) | 0 (0-0) | 0 (0-0) | 0 (0-0) | >0.99 | 0 (0-0) | 0 (0-0) | 0 (0-0) | 0 (0-0) | 0 (0-0) | >0.99 |
| **Necrosis, n (yes, %)** |  |  |  |  |  |  |  |  |  |  |  |  |  |  |  |  |  |  |
| **Supraosseous** | - | - | - | - | - | NA | - | - | - | - | - | NA | - | - | - | - | - | NA |
| **Intraosseous** | - | - | - | - | - | NA | - | - | - | - | - | NA | - | - | - | - | - | NA |
| **Active remodelling, n (yes, %)** |  |  |  |  |  |  |  |  |  |  |  |  |  |  |  |  |  |  |
| **Supraosseous** | 2 (33) | 2 (33) | - | - | - | 0.092 | 1 (12) | - | 2 (25) | - | - | 0.255 | 2 (17) | 3 (25) | - | - | - | 0.064 |
| **Intraosseous** | 2 (33) | 2 (33) | 2 (33) | - | - | 0.199 | 1 (12.5) | - | - | 1 (12.5) | - | 0.558 | - | 2 (17) | 1 (8) | - | - | 0.255 |
| **Periosteal reaction, n (%)** |  |  |  |  |  | NA |  |  |  |  |  | 0.776 |  |  |  |  |  | **0.001** |
| **None** | - | - | - | - | - |  | 1 (12) | - | 1 (12) | 2 (25) | - |  | - | - | - | - | 5 (42) |  |
| **Apposition** | 6 (100) | 6 (100) | 6 (100) | 6 (100) | 6 (100) |  | 7 (88) | 8 (100) | 7 (88) | 6 (75) | 6 (100) |  | 12 (100) | 12 (100) | 12 (100) | 12 (100) | 7 (58) |  |
| **Resorption** | - | - | - | - |  |  | - | - | - | - | - |  | - | - | - | - |  |  |
| **Endosteal reaction, n (%)** |  |  |  |  |  | >0.99 |  |  |  |  |  | >0.99 |  |  |  |  |  | >0.99 |
| **None** | 6 (100) | 6 (100) | 6 (100) | 5 (83) | 6 (100) |  | 8 (100) | 7 (88) | 8 (100) | 7 (88) | 8 (100) |  | 12 (100) | 12 (100) | 12 (100) | 12 (100) | 11 (92) |  |
| **Apposition** | - | - | - | 1 (17) | - |  | - | 1 (12) | - | 1 (12) | - |  | - | - | - | - | 1 (8) |  |
| **Resorption** | - | - | - | - | - |  | - | - | - | - | - |  | - | - | - | - | - |  |
| **Birefringent particles at non-implant site, n (yes, %)** | 1 (17) | 3 (50) | - | - | - | 0.075 | 1 (13) | - | 1 (13) | - | - | 0.406 | 2 (17) | 3 (25) | 3 (25) | 1 (8) | - | 0.339 |
| **Location** | Fibrous tissue | Bone and medulla | NA | NA | NA |  | Medulla | NA | Medulla | NA | NA |  | Bone and medulla | Bone and medulla | Bone and medulla | Bone and medulla | NA |  |
| **Type of cells** | Macrophage | Osteocytes and adipocytes | NA | NA | NA |  | Adipocytes | NA | Adipocytes | NA | NA |  | Adipocytes | Adipocytes | Adipocytes | Adipocytes | NA |  |

^*^ Data presented as median (25^th^-75^th^ percentile). ^#^Data presented as mean±SEM. Pairwise comparison with Bonferroni correction for multiple testing: ^a^P=0.050, ^b^P=0.019, ^c^P=0.018, ^d^P=0.045, ^e^P= 0.034, ^f^P=0.023, and ^g^P=0.004. Bold P-values are statistically significant (i.e. P<0.05). MNGCs, multinucleated giant cells; PMNs, polymorphonuclear leukocytes ; -: none (0.0%); NA: not applicable; SEM: standard error of the mean.

**Supplemental Table S7.** Multilevel models of each outcome scoring item at the supraosseous zone.

| **Outcome variable** | **Fragmentation score (n = 168)** | | | **Resorption score (n = 168)** | | | **Percentage new bone formation at supraosseous site (n = 168)** | | |
| --- | --- | --- | --- | --- | --- | --- | --- | --- | --- |
| **Model variable** | **β (95% CI)** | **OR (95% CI)^a^** | **P-value^b^** | **β (95% CI)** | **OR (95% CI)^a^** | **P-value^b^** | **β (95% CI)** | **OR (95% CI)^a^** | **P-value^b^** |
| Intercept | NA | NA | NA | NA | NA | NA | 19.44 (3.61;35.28) | NA | **0.016** |
| Osteosynthesis system (ref. = LactoSorb) |  |  | **<0.001^b^** |  |  | **<0.001^b^** |  |  | **<0.001^b^** |
| BioSorb FX | -1.31 (-2.62;0.01) | 0.27 0.073;1.00)^c^ | 0.051 | -1.25 (-2.88;0.38) | 0.29 (0.06;1.46)^c^ | 0.132 | -32.47 (-48.42;-16.52) | NA | **<0.001** |
| Inion CPS | -1.21 (-2.10;-0.32) | 0.30 (0.12;0.73)^c^ | **0.008** | -1.24 (-2.29;-0.19) | 0.29 (0.10;0.83)^c^ | **0.021** | -41.61 (-57.57;-25.66) | NA | **<0.001** |
| SonicWeld Rx | 4.13 (3.30;4.96) | 62.08 (27.0;146.7)^c^ | **<0.001** | 4.31 (3.13;5.48) | 74.05  (22.9;239.8) ^c^ | **<0.001** | 7.13  (-8.82;23.08) | NA | 0.379 |
| Follow-up (months) | 0.10 (0.08;0.13) | 1.11 (1.08;1.14)^c^ | **<0.001** | 0.11 (0.08;0.14) | 1.12 (1.08;1.15)^c^ | **<0.001** | 3.85 (2.72;4.98) | NA | **<0.001** |
| Follow-up^2^ (months^2^) | NA | NA | NA | NA | NA | NA | -0.05 (-0.07;-0.03) |  | **<0.001** |
| Osteosynthesis system * Follow-up |  |  | **<0.001^b^** |  |  | **<0.001^b^** |  |  | **<0.001^b^** |
| BioSorb FX | 0.01 (-0.04;0.06) | 1.01 (0.96;1.06)^c^ | 0.673 | 0.01 (-0.05;0.06) | 1.01 (0.96;1.06)^c^ | 0.850 | 0.60 (0.11;1.09) | NA | **0.018** |
| Inion CPS | 0.01 (-0.03;0.05) | 1.01 (0.97;1.05)^c^ | 0.652 | 0.01 (-0.03;0.05 | 1.01 (0.97;1.05)^c^ | 0.691 | 0.54 (0.04;1.03) | NA | **0.033** |
| SonicWeld Rx | -0.10 (-0.13;-0.08) | 0.90 (0.88;0.93)^c^ | **<0.001** | -0.11 (-0.14;-0.08) | 0.90 (0.87;0.93)^c^ | **<0.001** | -0.36 (-0.86;0.13) | NA | 0.149 |
| **Outcome variable** | **Type of new bone formation (n = 168)** | | | **Thickness of fibrous capsule (n = 168)** | | | **MNGCs at interface score (n = 168)** | | |
| **Model variables** | **β (95% CI)** | **OR (95% CI)^a^** | **P-value^b^** | **β (95% CI)** | **OR (95% CI)^a^** | **P-value^b^** | **β (95% CI)** | **OR (95% CI)^a^** | **P-value^b^** |
| Intercept | 0.25 (-0.90;1.40) | 1.29 (0.41;4.07)^d^ | 0.64 | NA | NA | NA | NA | NA | NA |
| Osteosynthesis system (ref. = LactoSorb) |  |  | **<0.001^b^** |  |  | 0.232^b^ |  |  | **0.006^b^** |
| BioSorb FX | -1.00 (-2.58;0.57) | 0.37 (0.08;1.77)^d^ | 0.211 | 1.18 (-0.27;2.62) | 3.35 (0.77;13.75)^c^ |  | -1.88 (-4.49;0.73) | 0.15 (0.01;2.08)^c^ | 0.157 |
| Inion CPS | -1.24 (-2.47;-0.01) | 0.29 (0.09;0.99)^d^ | **0.049** | 1.29 (-0.11;2.68) | 3.62 (0.89;14.65)^c^ |  | 0.02 (-1.45;1.49) | 1.02 (0.23;4.45)^c^ | 0.979 |
| SonicWeld Rx | 0.91 (-0.70;2.52) | 2.48 (0.50;12.38)^d^ | 0.265 | 0.25 (-1.05;1.55) | 1.29 (0.35;4.70)^c^ |  | 2.05 (-1.41;5.52) | 7.78 (0.24;248.27)^c^ | 0.244 |
| Follow-up (months) | 0.10 (0.06;0.15) | 1.11 (1.06;1.16)^d^ | **<0.001** | -0.11 (-0.14;-0.09) | 0.90 (0.87;0.92)^c^ | **<0.001** | -0.20 (-0.37;-0.02) | 0.82 (0.69;0.98)^c^ | **<0.001** |
| Osteosynthesis system * Follow-up |  |  | **<0.001^b^** |  |  | NA |  |  | **<0.001^b^** |
| BioSorb FX | 0.02 (-0.04;0.07) | 1.02 (0.96;1.07)^d^ | 0.605 | NA | NA | NA | 0.16 (0.02;0.30) | 1.18 (1.02;1.35)^c^ | **0.023** |
| Inion CPS | 0.03 (-0.08;0.02) | 1.03 (1.00;1.07)^d^ | 0.080 | NA | NA | NA | 0.06 (-0.04;0.15) | 1.06 (0.96;1.17)^c^ | 0.272 |
| SonicWeld Rx | -0.03 (-0.08;0.02) | 0.97 (0.92;1.02)^d^ | 0.188 | NA | NA | NA | -0.21 (-0.46;0.04) | 0.81 (0.631.04)^c^ | 0.094 |
| **Outcome variable** | **PMNs at interface score (n = 168)** | | | **Eosinophils at interface score (n = 168)** | | | **Adipocytes with birefringent particles at interface score (n = 168)** | | |
| **Model variables** | **β (95% CI)** | **OR (95% CI)^a^** | **P-value^b^** | **β (95% CI)** | **OR (95% CI)^a^** | **P-value^b^** | **β (95% CI)** | **OR (95% CI)^a^** | **P-value^b^** |
| Intercept | NA | NA | NA | NA | NA | NA | **None observed in all samples** | | |
| Osteosynthesis system (ref. = LactoSorb) |  |  | 0.621^b^ |  |  | 0.473**^b^** |  |  |  |
| BioSorb FX | -0.29 (-1.23;0.66) | 0.75 (0.29;1.93)^c^ |  | -0.40 (-1.49;0.69) | 0.67 (0.23;2.00)^c^ |  |  |  |  |
| Inion CPS | -0.29 (-1.23;0.66) | 0.75 (0.29;1.93)^c^ |  | -0.40 (-1.49;0.69) | 0.67 (0.23;2.00)^c^ |  |  |  |  |
| SonicWeld Rx | 0.07 (-1.36;1.50) | 1.07 (0.26;4.48)^c^ |  | -0.40 (-1.49;0.69) | 0.67 (0.23;2.00)^c^ |  |  |  |  |
| Follow-up (months) | -0.01 (-0.04;0.01) | 0.99 (0.96;1.01)^c^ | 0.300 | -0.01 (-0.03;0.01) | 0.99 (0.97;1.01)^c^ | 0.516 |  |  |  |
| **Outcome variable** | **Macrophages at interface score (n = 168)** | | | **Lymphocytes at interface score (n = 168)** | | | **Necrosis (n = 210)** | | |
| **Model variables** | **β (95% CI)** | **OR (95% CI)^a^** | **P-value^b^** | **β (95% CI)** | **OR (95% CI)^a^** | **P-value^b^** | **β (95% CI)** | **OR (95% CI)^a^** | **P-value^b^** |
| Osteosynthesis system (ref. = LactoSorb) |  |  | **0.020^b^** |  |  | 0.615**^b^** | **None observed in all samples** | | |
| BioSorb FX | -0.56 (-1.92;0.78) | 0.57 (0.15;2.21)^c^ | 0.413 | -0.36 (-1.29;0.58) | 0.70 (0.27;1.79)^c^ |  |  |  |  |
| Inion CPS | 0.74 (-1.13;2.61) | 2.10 (0.32;13.63)^c^ | 0.436 | -0.36 (-1.29;0.58) | 0.70 (0.27;1.79)^c^ |  |  |  |  |
| SonicWeld Rx | 0.05 (-0.96;1.06) | 1.05 (0.38;2.87)^c^ | 0.925 | -0.07 (-1.50;1.36) | 0.93 (0.22;3.89)^c^ |  |  |  |  |
| Follow-up (months) | -0.03 (0.09;-0.06) | 0.97 (0.94;1.01)^c^ | 0.093 | -0.01 (-0.04;0.01) | 0.99 (0.96;1.01)^c^ | 0.307 |  |  |  |
| **Outcome variable** | **Active remodelling (n = 210)** | | | **Periosteal reaction score (n = 210)** | | |  |  |  |
| **Model variables** | **β (95% CI)** | **OR (95% CI)^a^** | **P-value^b^** | **β (95% CI)** | **OR (95% CI)^a^** | **P-value^b^** |  |  |  |
| Intercept | 2.21 (-0.37;4.79) | 9.11 (0.69;119.97)^e^ | 0.093 | 3.07 (1.55;4.59) | 21.55 (4.70;98.79)^f^ | **<0.001** |  |  |  |
| Osteosynthesis system (ref. = LactoSorb) |  |  | **<0.001^b^** |  |  | **<0.001^b^** |  |  |  |
| Negative control | -18.79 (-20.87;-16.71) | 0.00 (0.00;0.00)^e^ | **<0.001** | -0.69 (-2.35;0.97) | 0.50 (0.10;2.63)^f^ | 0.411 |  |  |  |
| BioSorb FX | -2.96 (-5.61;-0.31) | 0.05 (0.00;0.74)^e^ | **0.029** | 0.64 (-0.38;1.66) | 1.90 (0.68;5.28)^f^ | 0.219 |  |  |  |
| Inion CPS | -3.96 (-6.17;-1.76) | 0.02 (0.00;0.17)^e^ | **<0.001** | 1.04 (0.07;2.00) | 2.82 (1.07;7.40)^f^ | **0.036** |  |  |  |
| SonicWeld Rx | -1.44 (-3.80;0.93) | 0.24 (0.02;2.53)^e^ | 0.232 | 0.30 (-0.40;1.00) | 1.35 (0.67;2.71)^f^ | 0.401 |  |  |  |
| Follow-up (months) | -0.25 (-0.43;-0.08) | 0.78 (0.65;0.92) | **0.027** | -0.02 (-0.05;0.01) | 0.98 (0.96;1.01)^f^ | 0.122 |  |  |  |
| Osteosynthesis system * Follow-up |  |  | **0.019^b^** |  |  | NA |  |  |  |
| Negative control | 0.25 (0.09;0.41) | 1.28 (1.09;1.51)^e^ | **0.003** | NA | NA | NA |  |  |  |
| BioSorb FX | 0.24 (0.08;0.39) | 1.27 (1.09;1.47)^e^ | **0.002** | NA | NA | NA |  |  |  |
| Inion CPS | 0.26 (0.11;0.42) | 1.30 (1.11;1.51)^e^ | **0.001** | NA | NA | NA |  |  |  |
| SonicWeld Rx | 0.16 (-0.02;0.33) | 1.17 (0.98;1.40)^e^ | 0.077 | NA | NA | NA |  |  |  |

^a^Calculated in case of binary or ordinal outcome variables; ^b^P-value of the complete block; ^c^Odds ratio of having a higher score of that specific item; ^d^Odds ratio of formation of lamellar bone compared to no new bone formation; ^e^Odds ratio of the presence of active remodelling; ^f^Odds ratio of the presence of periosteal reaction.

Abbreviations: β, estimated coefficient; CI, confidence interval; OR, odds ratio; NA, not applicable; Ref, reference; MNGCs, multinucleated giant cells; PMNs: polymorphonuclear leukocytes.

**Supplemental Table S8.** Multilevel models of each outcome scoring item at the intraosseous zone and at non-implant site.

| **Outcome variable** | **Fragmentation score (n = 168)** | | | **Resorption score (n = 168)** | | | **Percentage new bone formation at intraosseous site**  **(n = 168)** | | |
| --- | --- | --- | --- | --- | --- | --- | --- | --- | --- |
| **Model variables** | **β (95% CI)** | **OR (95% CI)^a^** | **P-value^b^** | **β (95% CI)** | **OR (95% CI)^a^** | **P-value^b^** | **β (95% CI)** | **OR (95% CI)^a^** | **P-value^b^** |
| Intercept | NA | NA | NA | NA | NA | NA | 11.43 (-6.77;29.64) | NA |  |
| Osteosynthesis system (ref. = LactoSorb) |  |  | **<0.001^b^** |  |  | **<0.001^b^** |  | NA | **<0.001^b^** |
| BioSorb FX | -0.41 (-1.57;0.74) | 0.66 (0.21;2.10)^c^ | 0.482 | 2.18 (1.05;3.30) | 8.83 (2.87;27.18)^c^ | **<0.001** | -28.27 (-45.51;-11.04) | NA | **0.001** |
| Inion CPS | -0.23 (-1.24;0.79) | 0.80 (0.29;2.20)^c^ | 0.660 | 1.21 (-1.61;4.02) | 3.34 (0.20;55.73)^c^ | 0.399 | -30.95 (-48.19;-13.72) | NA | **0.001** |
| SonicWeld Rx | 4.71 (3.54;5.89) | 111.13 (34.32;359.81)^c^ | **<0.001** | 24.22 (22.26;26.18) | 33*10^9^ (46*10^8^;23*10^10^)^#,c^ | **<0.001** | 11.36 (-5.87;28.60) | NA | 0.195 |
| Follow-up (months) | 0.12 (0.08;0.16) | 1.13 (1.09;1.17)^c^ | **<0.001** | 0.45 (0.25;0.64) | 1.56 (1.29;1.90) ^c^ | **<0.001** | 4.74 (3.40;6.09) | NA | **<0.001** |
| Follow-up^2^ (months^2^) | NA | NA | **NA** | NA | NA | NA | -0.07 (-0.09;-0.04) | NA | **<0.001** |
| Osteosynthesis system * Follow-up |  |  | **<0.001^b^** |  |  | **<0.001^b^** |  | NA | **0.001^b^** |
| BioSorb FX | -0.01 (-0.05;0.03) | 0.99 (0.95;1.03)^c^ | 0.671 | -0.28 (-0.45;-0.11) | 0.76 (0.64;0.90)^c^ | **0.001** | 0.39 (-0.14;0.93) | NA | 0.149 |
| Inion CPS | -0.00 (-0.03;0.03) | 1.00 (0.97;1.03)^c^ | 0.949 | -0.15 (-0.43;0.13) | 0.86 (0.65;1.14)^c^ | 0.286 | 0.54 (0.00;1.07) | NA | **0.049** |
| SonicWeld Rx | -0.12 (-0.16;-0.08) | 0.89 (0.85;0.92)^c^ | **<0.001** | -0.46 (-0.65;-0.28) | 0.63 (0.52;0.76)^c^ | **<0.001** | -0.48 (-1.01;0.06) | NA | 0.080 |
| **Outcome variable** | **Type of new bone formation (n = 168)** | | | **Thickness of fibrous capsule (n = 168)** | | | **MNGCs at interface score (n = 168)** | | |
| **Model variables** | **β (95% CI)** | **OR (95% CI)^a^** | **P-value^b^** | **β (95% CI)** | **OR (95% CI)^a^** | **P-value^b^** | **β (95% CI)** | **OR (95% CI)^a^** | **P-value^b^** |
| Intercept | -2.02 (-3.98;-0.06) | 0.13 (0.02;0.94) | **0.043** | NA | NA | NA | NA | NA | NA |
| Osteosynthesis system (ref. = LactoSorb) |  |  | 0.832^b^ |  |  | 0.093^b^ |  |  | **<0.001^b^** |
| BioSorb FX | 0.47 (-1.42;2.36) | 1.59 (0.24;10.54)^d^ |  | 0.97 (0.08;1.87) | 2.65 (1.09;6.46)^c^ |  | 1.60 (-0.78;3.97) | 4.93 (0.46;53.16)^c^ | 0.187 |
| Inion CPS | -0.42 (-2.21;1.37) | 0.66 (0.11;3.95)^d^ |  | 1.13 (0.20;2.05) | 3.08 (1.23;7.75)^c^ |  | 0.83 (-0.87;2.53) | 2.30 (0.42;12.53)^c^ | 0.335 |
| SonicWeld Rx | 0.00 (-1.82;1.82) | 1.00 (0.16;6.18)^d^ |  | 0.22 (-0.66;1.09) | 1.24 (0.52;2.99)^c^ |  | 0.62 (0.45;0.78) | 1.86 (1.58;2.19)^c^ | **<0.001** |
| Follow-up (months) | 0.32 (0.14;0.49) | 1.37 (1.16;1.63)^d^ | **<0.001** | -0.11 (-0.13;-0.09) | 0.90 (0.88;0.92)^c^ | **<0.001** | -0.10 (-0.15;-0.05) | 0.90 (0.86;0.95)^c^ | **<0.001** |
| Osteosynthesis system * Follow-up |  |  | NA |  |  | NA |  |  | NA |
| BioSorb FX | NA | NA | NA | NA | NA | NA | NA | NA | NA |
| Inion CPS | NA | NA | NA | NA | NA | NA | NA | NA | NA |
| SonicWeld Rx | NA | NA | NA | NA | NA | NA | NA | NA | NA |
| **Outcome variable** | **PMNs at interface score (n = 168)** | | | **Eosinophils at interface score (n = 168)** | | | **Adipocytes with birefringent particles at interface score (n = 168)** | | |
| **Model variables** | **β (95% CI)** | **OR (95% CI)^a^** | **P-value^b^** | **β (95% CI)** | **OR (95% CI)^a^** | **P-value^b^** | **β (95% CI)** | **OR (95% CI)^a^** | **P-value^b^** |
| Intercept | NA | NA | NA | **None observed in all samples** | | | **None observed in all samples** | | |
| Osteosynthesis system (ref. = LactoSorb) |  |  | 0.473^b^ |  |  |  |  |  |  |
| BioSorb FX | -0.40 (-1.49;0.69) | 0.67 (0.23;2.00)^c^ |  |  |  |  |  |  |  |
| Inion CPS | -0.40 (-1.49;0.69) | 0.67 (0.23;2.00)^c^ |  |  |  |  |  |  |  |
| SonicWeld Rx | -0.40 (-1.49;0.69) | 0.67 (0.23;2.00)^c^ |  |  |  |  |  |  |  |
| Follow-up (months) | -0.01 (-0.03;0.01) | 0.99 (0.97;1.01)^c^ | 0.516 |  |  |  |  |  |  |
| **Outcome variable** | **Macrophages at interface score (n = 168)** | | | **Lymphocytes at interface score (n = 168)** | | | **Necrosis (n = 210)** | | |
| **Model variables** | **β (95% CI)** | **OR (95% CI)^a^** | **P-value^b^** | **β (95% CI)** | **OR (95% CI)^a^** | **P-value^b^** | **β (95% CI)** | **OR (95% CI)^a^** | **P-value^b^** |
| Osteosynthesis system (ref. = LactoSorb) |  |  | 0.981^b^ |  |  | 0.477**^b^** | **None observed in all samples** | | |
| BioSorb FX | 0.00 (-2.58;2.58) | 1.00 (0.08;13.16)^c^ |  | 0.40 (-0.70;1.49) | 1.49 (0.50;4.45)^c^ |  |  |  |  |
| Inion CPS | 0.40 (1.97;2.77) | 1.49 (0.14;15.90)^c^ |  | 0.00 (0.00;0.00) | 1.00 (1.00;1.00)^c^ |  |  |  |  |
| SonicWeld Rx | 0.00 (-2.58;2.58) | 1.00 (0.08;13.16)^c^ |  | 0.00 (0.00;0.00) | 1.00 (1.00;1.00)^c^ |  |  |  |  |
| Follow-up (months) | -0.00 (-0.06;0.05) | 1.00 (0.94;1.05)^c^ | 0.878 | 0.00 (-0.02;0.01) | 0.97 (0.98;1.01)^c^ | 0.571 |  |  |  |
| **Outcome variable** | **Active remodelling (n = 210)** | | | **Endosteal reaction score (n = 210)** | | | **Percentage new bone formation at complete implant site**  **(n = 210)** | | |
| **Model variables** | **β (95% CI)** | **OR (95% CI)^a^** | **P-value^b^** | **β (95% CI)** | **OR (95% CI)^a^** | **P-value^b^** | **β (95% CI)** | **OR (95% CI)^a^** | **P-value^b^** |
| Intercept | NA | NA | NA | NA | NA | NA | 24.31 (11.00;37.60) | NA | **<0.001** |
| Osteosynthesis system (ref. = LactoSorb) |  |  | 0.525^b^ |  |  | **<0.001^b^** |  | NA | **<0.001^b^** |
| Negative control | -15.53 (-1915;1884) | 0.00 (0.00;†)^e^ |  | -0.90 (-2.77;0.97) | 0.41 (0.06;2.63)^f^ |  | 46.46 (32.45;60.46) | NA | **<0.001** |
| BioSorb FX | 0.42 (0.87;1.70) | 1.52 (0.42;5.46)^e^ |  | 2.57 (-0.01;5.16) | 13.09 (0.99;173.51)^f^ |  | -32.27 (-46.28;-18.27) | NA | **<0.001** |
| Inion CPS | 0.42 (0.87;1.70) | 1.52 (0.42;5.46)^e^ |  | 1.67 (-0.87;4.22) | 5.33 (0.42;67.85)^f^ |  | -39.60 (-53.61;-25.60) | NA | **<0.001** |
| SonicWeld Rx | 1.04 (-0.17;2.25) | 2.83 (0.84;0.95)^e^ |  | -0.84 (-2.73;1.06) | 0.43 (0.07;2.87)^f^ |  | 7.50 (-6.51;21.50) | NA | 0.292 |
| Follow-up (months) | -0.05 (-0.08;-0.01) | 0.95 (0.92;0.99)^e^ | **0.011** | 0.01 (-0.04;0.05) | 1.01 (0.96;1.06)^f^ | 0.807 | 3.45 (2.54;4.37) | NA | **<0.001** |
| Follow-up^2^ (months^2^) | NA | NA | NA | NA | NA | NA | -0.04 (-0.06;-0.03) | NA | **<0.001** |
| Osteosynthesis system * Follow-up |  |  | NA |  |  | **<0.001^b^** |  | NA | **<0.001^b^** |
| Negative control |  |  |  | -0.01 (-0.05;0.04) | 1.00 (0.95;1.05)^f^ | 0.843 | -1.09 (-1.52;-0.65) | NA | **<0.001** |
| BioSorb FX | NA | NA | NA | -0.09 (-0.17;-0.01) | 0.91 (0.84;0.99)^f^ | **0.024** | 0.58 (0.15;1.01) | NA | **0.009** |
| Inion CPS | NA | NA | NA | -0.05 (-0.14;0.04) | 0.95 (0.87;1.04)^f^ | 0.252 | 0.58 (0.14;1.01) | NA | **0.009** |
| SonicWeld Rx | NA | NA | NA | -0.01 (-0.06;0.04) | 0.99 (0.95;1.04)^f^ | 0.772 | -0.37 (-0.80;0.07) | NA | 0.095 |
| **Outcome variable** | **Distant MNGCs with birefringent particles**  **(n = 210)** | | | **Distant PMNs with birefringent particles**  **(n = 210)** | | | **Distant eosinophils with birefringent particles**  **(n = 210)** | | |
| **Model variables** | **β (95% CI)** | **OR (95% CI)^a^** | **P-value^b^** | **β (95% CI)** | **OR (95% CI)^a^** | **P-value^b^** | **β (95% CI)** | **OR (95% CI)^a^** | **P-value^b^** |
| Osteosynthesis system (ref. = LactoSorb) |  |  | 0.995**^b^** | **None observed in all samples** | | | **None observed in all samples** | | |
| Negative control | 0.00 (-2.59;2.59) | 1.00 (0.08;13.32)^c^ |  |  |  |  |  |  |  |
| BioSorb FX | 0.00 (-2.59;2.59) | 1.00 (0.08;13.32)^c^ |  |  |  |  |  |  |  |
| Inion CPS | 0.40 (-1.99;2.78) | 1.49 (0.14;16.12)^c^ |  |  |  |  |  |  |  |
| SonicWeld Rx | 0.00 (-2.59;2.59) | 1.00 (0.08;13.32)^c^ |  |  |  |  |  |  |  |
| Follow-up (months) | -0.01 (-0.06;0.05) | 0.99 (0.95;1.05)^c^ | 0.828 |  |  |  |  |  |  |
| **Outcome variable** | **Distant adipocytes with birefringent particles (n = 210)** | | | **Distant macrophages with birefringent particles (n = 210)** | | | **Distant lymphocytes with birefringent particles (n = 210)** | | |
| **Model variables** | **β (95% CI)** | **OR (95% CI)^a^** | **P-value^b^** | **β (95% CI)** | **OR (95% CI)^a^** | **P-value^b^** | **β (95% CI)** | **OR (95% CI)^a^** | **P-value^b^** |
| Osteosynthesis system (ref. = LactoSorb) |  |  | 0.437**^b^** |  |  | 0.947^b^ | **None observed in all samples** | | |
| Negative control | -14.42 (-2605;2576) | 0.00 (0.00;&)^c^ |  | 0.00 (-2.34;2.34) | 1.00 (0.10;10.41)^c^ |  |  |  |  |
| BioSorb FX | 1.59 (-0.74;3.92) | 4.90 (0.48;50.47)^c^ |  | 0.29 (-1.91;2.49) | 1.34 (0.15;12.09)^c^ |  |  |  |  |
| Inion CPS | 1.66 (-0.66;3.98) | 5.25 (0.52;53.53)^c^ |  | 0.67 (-1.39;2.73) | 1.96 (0.25;15.40)^c^ |  |  |  |  |
| SonicWeld Rx | -0.01 (-2.91;2.89) | 0.99 (0.06;18.01)^c^ |  | 0.00 (-2.34;2.34) | 1.00 (0.10;10.41)^c^ |  |  |  |  |
| Follow-up (months) | -0.01 (-0.08;0.06) | 0.99 (0.93;1.07)^c^ | 0.842 | -0.01 (-0.05;0.03) | 0.99 (0.95;1.03)^c^ | 0.630 |  |  |  |
| Osteosynthesis system * Follow-up |  |  | NA |  |  | NA |  |  |  |
| BioSorb FX | NA | NA | NA | NA | NA | NA |  |  |  |
| Inion CPS | NA | NA | NA | NA | NA | NA |  |  |  |
| SonicWeld Rx | NA | NA | NA | NA | NA | NA |  |  |  |
| **Outcome variable** | **Distant osteocytes with birefringent particles**  **(n = 210)** | | | **Birefringent particles at non-implant site**  **(n = 210)** | | |  |  |  |
| **Model variables** | **β (95% CI)** | **OR (95% CI)^a^** | **P-value^b^** | **β (95% CI)** | **OR (95% CI)^a^** | **P-value^b^** |  |  |  |
| Intercept | NA | NA | NA | -1.75 (-3.87;0.36) | 0.17 (0.02;1.44)^g^ | 0.104 |  |  |  |
| Osteosynthesis system (ref. = LactoSorb) |  |  | 0.838**^b^** |  |  | **<0.001^b^** |  |  |  |
| Negative control | -0.50 (-2.53;1.53) | 0.61 (0.08;4.61)^c^ |  | -15.75 (-17.77;-13.73) | 0.00 (0.00;0.00)^g^ | **<0.001** |  |  |  |
| BioSorb FX | -0.50 (-2.53;1.53) | 0.61 (0.08;4.61)^c^ |  | -0.78 (-4.21;2.65) | 0.45 (0.02;14.18)^g^ | 0.655 |  |  |  |
| Inion CPS | 0.35 (-1.33;2.02) | 1.42 (0.27;7.56)^c^ |  | -0.13 (-3.16;2.90) | 0.88 (0.04;18.16)^g^ | 0.933 |  |  |  |
| SonicWeld Rx | -0.50 (-2.53;1.53) | 0.61 (0.08;4.61)^c^ |  | -5.10 (-8.90;-1.29) | 0.01 (0.00;0.28)^g^ | **0.009** |  |  |  |
| Follow-up (months) | -0.01 (-0.05;0.03) | 0.99 (0.95;1.03)^c^ | 0.503 | -0.03 (-0.05;0.11) | 0.97 (0.88;1.06)^g^ | 0.227 |  |  |  |
| Osteosynthesis system * Follow-up |  |  | NA |  |  | **0.008^b^** |  |  |  |
| Negative control | NA | NA | NA | 0.03 (-0.05;0.11) | 1.03 (0.95;1.12)^g^ | 0.457 |  |  |  |
| BioSorb FX | NA | NA | NA | 0.05 (-0.06;0.16) | 1.05 (0.94;1.17)^g^ | 0.367 |  |  |  |
| Inion CPS | NA | NA | NA | 0.05 (-0.04;0.13) | 1.05 (0.96;1.14)^g^ | 0.277 |  |  |  |
| SonicWeld Rx | NA | NA | NA | 0.15 (0.06;0.25) | 1.17 (1.06;1.28)^g^ | **<0.001** |  |  |  |

^a^Calculated in case of binary or ordinal outcome variables; ^b^P-value of the complete block; ^c^Odds ratio of having a higher score of that specific item; ^d^Odds ratio of formation of lamellar bone compared to no new bone formation; ^e^Odds ratio of the presence of active remodelling; ^f^Odds ratio of the presence of endosteal reaction; ^g^Odds ratio of the presence of birefringent particles at non-implant site; ^#^Very large coefficient and, thus, odds ratio due to that this osteosynthesis system scored ‘complete resorption’ in all samples (i.e., no observed variance); ^†^Very large upper limit of the 95% CI due to that none of the negative control samples showed active remodeling; ^&^Very large upper limit of the 95% CI due to that none of the negative control samples showed distant adipocytes with birefringent particles.

Abbreviations: β, estimated coefficient; CI, confidence interval; OR, odds ratio; Ref, reference group; NA, not applicable; MNGCs, multinucleated giant cells; PMNs, polymorphonuclear leukocytes


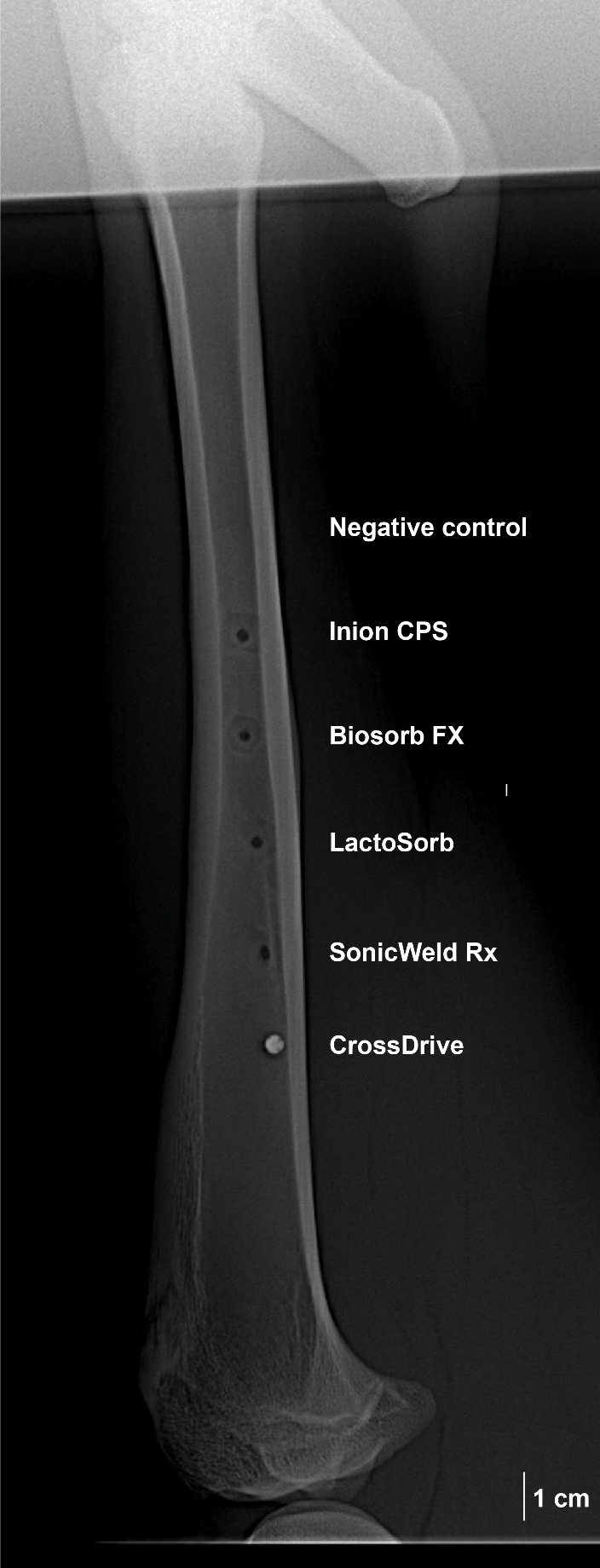


**Supplemental Figure S1.** X-ray radiographs of the surgical sites at 6-months follow-up

**
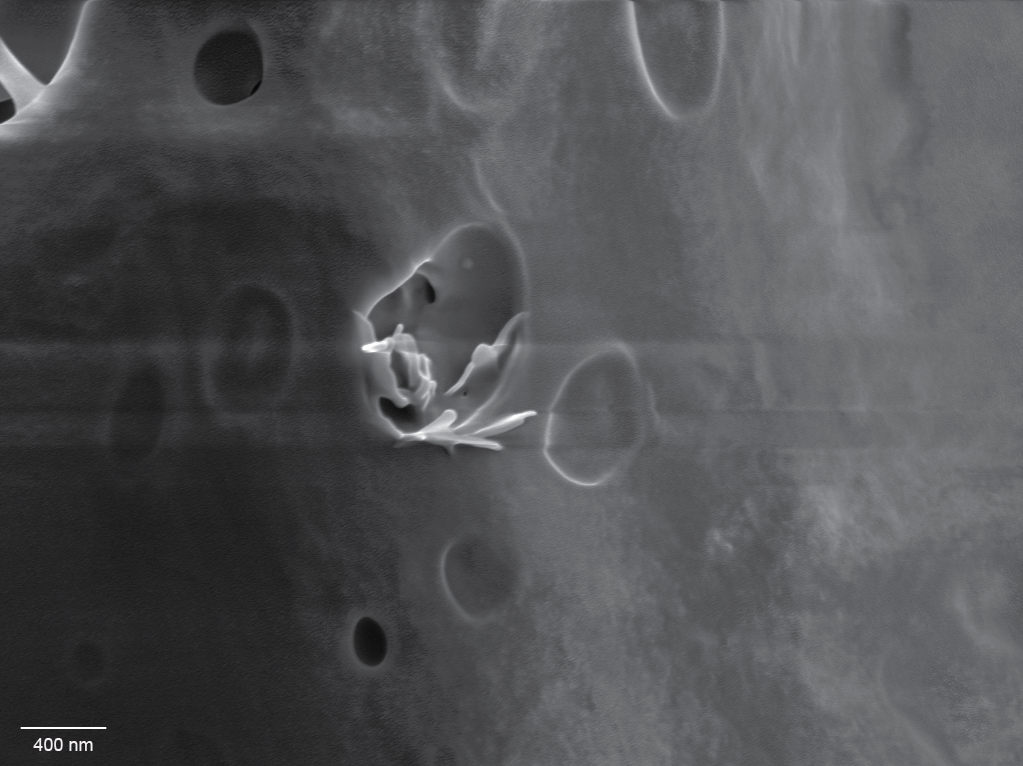
**

**Supplemental Figure S2.** Scanning electron microscopy image of crystalline, needle-like structures of nanoscale in randomly selected vacuoles of the medulla after implantation of the BioSorb FX system at 36 months follow-up (magnification 52.860x).
